# Ion Crowding Effect in Unilaterally Downsized Perovskite Memristors

**DOI:** 10.1002/advs.202524258

**Published:** 2026-02-04

**Authors:** Conghui Tan, Meiqi An, Weili Liu, Haotian Wang, Shuai Yang, Jing Li, Jianliang Li, Yingmin Luo, Chao Wang, Jiao Xu, Yaodan Chi, Yiming Yang

**Affiliations:** ^1^ School of Integrated Circuits Dalian University of Technology Dalian China; ^2^ School of Instrument and Electronics North University of China Taiyuan China; ^3^ Key Laboratory of Materials Modification by Laser, Ion, and Electron Beams (Ministry of Education) School of Physics Dalian University of Technology Dalian China; ^4^ Key Laboratory of Architectural Cold Climate Energy Management Ministry of Education Jilin Jianzhu University Changchun China

**Keywords:** conductive atomic force microscope, halide perovskites, ionic transport, nanomaterials, resistive switching

## Abstract

Minimizing the footprint of individual cells in a memristor array is crucial for increasing packing density, reducing power consumption and boosting computational performance. However, the downscaling of memristive devices incorporating halide perovskites has been challenging due to the polycrystalline nature of the active layer. In this work, we employed monocrystalline nanoplates of the all‐inorganic perovskite CsPbBr_3_, and combined nanofabrication and conductive atomic force microscopy to progressively downsize the memristors to the micrometer and nanometer scale. We report an ion crowding effect in these micro‐ and nano‐ devices with unilaterally downscaled electrodes. The ion crowding effect is analogous to the current crowding effect in bipolar junction transistors, and originates from the substantially enhanced electric field in the peripheral of downsized electrodes. This effect fundamentally alters the microscopic ionic transport and distribution, leading to distinct switching behaviors and morphological deformation including protrusions and indentations. Further downsizing the critical dimension of memristors to 30 nm intensifies the crowding effect, resulting in anisotropic switching characteristics and unique “hole‐in‐a‐bump” surface feature. This study offers insight into the field‐induced ionic behaviors at microscale, and lays the groundwork for miniaturized perovskite memristors.

## Graphical abstract

The downscaling of perovskite memristors incorporating monocrystalline nanoplates is accompanied by an ion crowding effect, which greatly enhances the local electric field in the vicinity of electrode. The highly controlled crowding of anions and vacancies results in anisotropic switching characteristics as well as unique morphology modification including protrusions, indentations, and “hole‐in‐a‐bump” surface features.

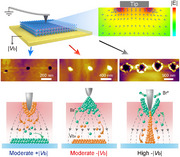

## 1 Introduction

**FIGURE 1:**
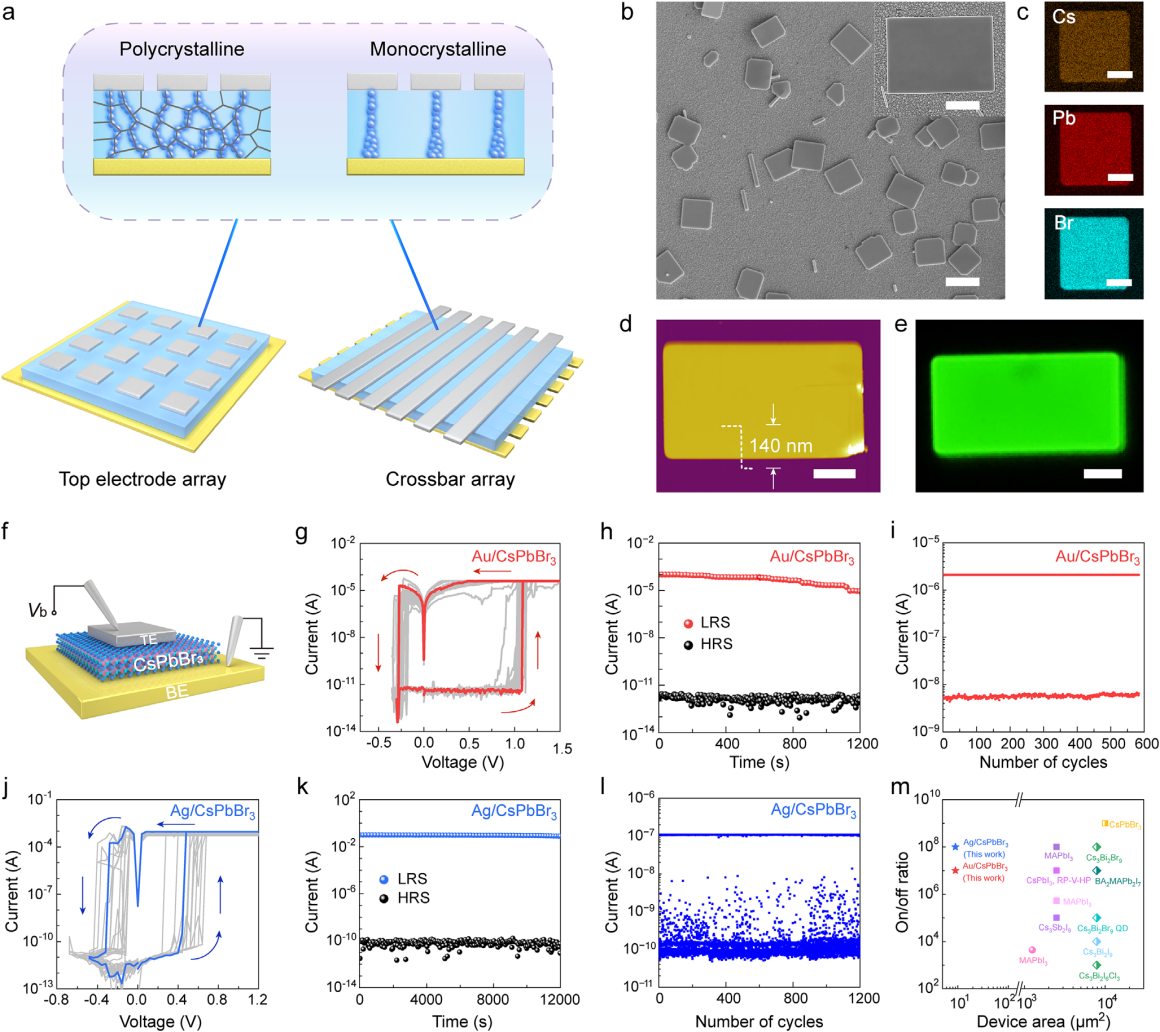
Performance of NP memristors with downsized electrodes. (a) Illustration of typical device architectures including TE array and crossbar array. The zoom‐in view shows the comparison of CFs in polycrystalline and monocrystalline devices. (b) SEM image of the CsPbBr_3_ NPs grown on a FTO substrate. Inset: magnified image of a single NP. (c) EDS elemental mappings of Cs, Pb, and Br in a single NP. (d) AFM image of a NP with thickness of 140 nm (white dashed lines). (e) PL image of a single NP. Scale bars: (b) 5 µm, inset: 1 µm, (c) 2 µm, (d) 1 µm, (e) 1 µm. (f) Schematic drawing of a sandwiched memristor with downsized TE dimension of 3 × 3 µm. (g) Multiple *I*–*V* sweeps of Au/CsPbBr_3_/Au with *I*_CC_ of 40 µA. The arrows denote the sweeping direction. The first sweep is marked red. (h) Retention characteristics of the Au/CsPbBr_3_ device under reading bias of 0.02 V. (i) Pulse endurance test of Au/CsPbBr_3_ memristor showing over 600 programming cycles. The applied set and read pulses are 7 V/2 ms and 0.02 V/6 ms, respectively. (j) Multiple *I*–*V* sweeps of Ag/CsPbBr_3_/Au under *I*_CC_ of 0.5 mA. The first sweep is marked blue. (k) Retention tests of the Ag/CsPbBr_3_/Au device under reading bias of 0.02 V. (l) Pulse endurance test of Ag/CsPbBr_3_/Au memristor showing over 1200 programming cycles. The applied set and read pulses are 1.5 V/2 ms and 0.05 V/6 ms, respectively. m) Comparison of on/off ratio and the device area among various perovskite memristors [19, 30, 37, 38, 39, 40, 41, 42, 43, 44, 45, 46].

Memristive devices have attracted widespread attention as a potential solution to the von Neumann bottleneck [1, 2], a promising candidate for artificial synapses [3, 4, 5, 6, 7], and a versatile contender with integration of opto‐sensing, data storage and processing [8, 9, 10, 11]. In many scenarios, arrayed architectures incorporating identical memristive units are required to achieve specific computational functions [12, 13, 14, 15]. Therefore, the downscaling of single memory cells directly determines the footprint, packing density, performance, and energy consumption of the entire array. To reduce the size of individual memristors, it is often feasible to only downsize the electrodes, since these arrays often adopt the vertical sandwiched cross‐bar structure [16, 17, 18] as well as top electrode (TE) arrays with a shared bottom electrode (Figure 1a) [19, 20, 21, 22]. Currently, in state‐of‐the‐art memristor arrays, the critical dimension (CD) of individual devices has been downsized to only a few nanometers [23, 24], translating to a tremendously high information density of terabits per square inch. Nevertheless, to accommodate the need for multi‐scenario applications, extra features including photosensitivity, wearability, neuromorphic computing capability of devices are also required in addition to their memristive properties.

As an emerging class of semiconductors, metal halide perovskites (MHPs) possess encouraging advantages for multi‐functional memristors thanks to their highly mobile ions, excellent optoelectronic features and superior flexibility [25, 26, 27, 28, 29]. MHP‐based memristors with vacancy and metallic conductive filaments (CFs) have both been demonstrated, exhibiting merits of low power consumption, decent flexibility, high on/off ratio, light‐induced switching, and synaptic behaviors [30, 31, 32, 33, 34, 35]. However, the downsizing of device has been formidable in MHP memristors, which is largely due to the uniformity of the active layer comprising solution‐processed MHP thin films. In a polycrystalline film, the size, boundaries, and orientation of grains critically affect the microscopic ionic behavior and subsequent memristive performance. For example, previous reports have shown that the grain boundaries facilitate ion migration and CF formation [36]. Thus, as the geometry of device approaches the grain size, the memristive properties are subject to device‐to‐device variations, and the homogeneity of the array cannot be guaranteed (Figure 1a). To date, existing MHP memristors mainly employ electrode dimensions spanning from tens of micrometers to millimeters (Table S1) [19, 30, 37, 38, 39, 40, 41, 42, 43, 44, 45, 46, 47, 48]. The validity and reliability of downsized MHP memristors, particularly those with CDs approaching and below micrometer‐level, are largely unexplored at the present. To investigate the downsizing capability, thin monocrystalline structures, exemplified by the micro‐ and nanoplates (NPs) of perovskites or the 2D Ruddlesden‐Popper and Dion‐Jacobson phase, offer a natural platform for downscaling of MHP memristors. These 2D crystals are experimentally available [49, 50, 51], while the downsizing of electrodes is feasible through nanofabrication or scanning‐probe techniques [52, 53, 54, 55, 56]. Moreover, considering the robustness of the all‐inorganic halide perovskites, the CsPbX_3_ (X = Cl, Br, and I) becomes one of the ideal systems for such study.

In this work, we fabricated various micro‐ and nanoscale memristive devices with downsized TEs in monocrystalline CsPbBr_3_ NPs. The moderately downscaled devices maintain the conventional resistive switching features with ultrahigh on/off ratio. However, as the CD is further reduced to approximately 1 µm, the set voltage of switching significantly increases, accompanied by apparent protrusions in the local morphology. We propose an ion crowding effect, which is analogous to the current crowding effect in electronic systems, to account for the observed phenomena. Due to a greatly enhanced local electrical field, the halogen anions or vacancies concentrate to the edge of TE and predominantly alter the memristor performance. By downsizing the CD to nanometer regime, the ion crowding effect is further enhanced through overlapping of the peripheral field, leading to unique morphological modifications and switching characteristics. Among complex ionic/electronic migration in the HP memristors, the ion crowding effect extends from electric field‐induced ion migration, which could dominate the resistive switching of soft lattice perovskite memristors at the nanoscale [57, 58]. These findings reveal the micro‐ and nanoscale behaviors of ions under intense electric field, and offer guidelines to minimize the footprint of perovskite memristors.

## 2 Results and Discussion

### 2.1 Downscaling Memristor Footprint to Micrometer Level

The thin CsPbBr_3_ NPs were synthesized on fluorine‐doped tin oxide (FTO) substrates via a solution recrystallization method (more details in Experimental Section). In a typical growth, large amount of square and rectangular shaped NPs were produced and monodispersed on the substrate, featuring lateral size ranging from 5 to 20 µm with smooth surface (Figure 1b). The X‐ray diffraction (XRD) pattern confirms the orthorhombic phase of CsPbBr_3_ with preferable orientation along the [001] direction (Figure S1). The elemental mappings from energy dispersive X‐ray spectroscopy (EDS) demonstrate uniform distribution of Cs, Pb, and Br (Figure 1c). The thickness of the NPs, determined by an atomic force microscope (AFM), ranges from 100 to 300 nm (Figures 1d; S2). The average surface roughness of a typical NP with thickness of 140 nm is below 0.15 nm (Figure S3). The micro‐photoluminescence (micro‐PL) image of an individual NP shows bright green‐colored emission (Figure 1e) with corresponding peak centering at 528 nm, consistent with the emission of bulk CsPbBr_3_. In addition, time‐resolved PL measurement of a single NP exhibits bi‐exponential decay with an average lifetime of 3.7 ns (Figure S4). These characterizations confirm the high crystallinity of the NPs, which offers the basis for subsequent study of resistive switching.

The as‐grown NPs were transferred by a micromanipulator onto a prepatterned gold bottom electrode (BE). To reduce the footprint of device, a nondestructive electron‐beam lithography was employed to define the area of TEs on a NP, followed by subsequent thermal evaporation of gold or silver. Detailed nanofabrication procedures are shown in the *Experimental Section* and Figure S5 (Supporting Information). A vertical sandwiched structure of TE/CsPbBr_3_/BE was employed (Figure 1f), in which the lateral size of TE was shrinked to a square of 3 µm × 3 µm. Compared to previous reports of thin‐film MHP memristors (Figure 1m and Table S1) [19, 30, 37, 38, 39, 40, 41, 42, 43, 44, 45, 46, 47, 48], the CD of device is downsized by over 1000‐fold. Next, the switching behavior of Au/CsPbBr_3_/Au device was evaluated through external electrical contacts with two microprobes (Figure 1f). Upon applying a positive bias to TE, the device demonstrates abrupt switching from the initial high resistance state (HRS) to a low resistance state (LRS) under compliance current (*I*_CC_) of 40 µA (Figure 1g). Notably, the resistive switching requires no electroforming process, and occurs during the first voltage sweep. By applying repetitive sweeps of 0 → 1.5 → 0 → ‐0.35 → 0 V, the device exhibits typical bipolar non‐volatile switching behavior. Over 25 switching cycles, the average set and reset voltages feature 1 V and ‐0.25 V, respectively (Figure S6). The HRS exhibits resistance of 10^12^ Ω, which is drastically reduced to LRS of 10^5^ Ω upon switching, leading to an ultrahigh on/off ratio of 10^7^. The retention test shows unchanged HRS and slightly decreased LRS over 10^3^ s (Figure 1h). Endurance tests demonstrate reproducible resistive switching behavior over 600 cycles and high uniformity at HRS for the Au/CsPbBr_3_ device (Figure 1i). Moreover, by replacing gold with silver for TE, the on/off ratio rises to 10^8^ with average set voltage decreasing to 0.5 V (Figures 1j; S7). The lower set voltage in Ag‐based devices can be attributed to the facile formation of silver CFs in MHPs rather than the vacancy CFs in Au‐based devices [28, 30, 59]. The retention of the Ag/CsPbBr_3_ device extends over 10^4^ s and the pulse endurance tests demonstrate over 1200 cycles (Figures 1k and 1l). Additionally, at lower *I*_CC_ between 1 µA and 1 mA, both devices deliver stable volatile threshold‐switching behavior (Figures S8 and S9). Compared to thin‐film MHP memristors, the downsized devices demonstrate one of the highest on/off ratios amongst reported work (Figure 1m; Table S1) [19, 30, 37, 38, 39, 40, 41, 42, 43, 44, 45, 46, 47, 48]. The ultrahigh on/off ratio might be attributed to the reduced defects in monocrystalline NPs and downscaling of memristor footprint, which substantially suppress the dark conduction current. Despite of a higher on/off ratio and lower set voltage, the long‐term robustness of the Ag/CsPbBr_3_ device is questionable, owing to the chemical reaction between silver and CsPbBr_3_ [60]. In the NP devices, we have observed spontaneous decay in the morphology of Ag/CsPbBr_3_ devices upon storage in nitrogen environment for 11 days (Figure S10), which may compromise their downsizing capabilities. In contrast, no decay was detected in the Au/CsPbBr_3_ device stored for one month in nitrogen (Figure S11). Overall, the micrometer‐sized devices generally demonstrate superior switching performance to conventional thin‐film memristors, and inert electrodes including gold or platinium may offer enhanced stability for the downscaled devices.

**FIGURE 2:**
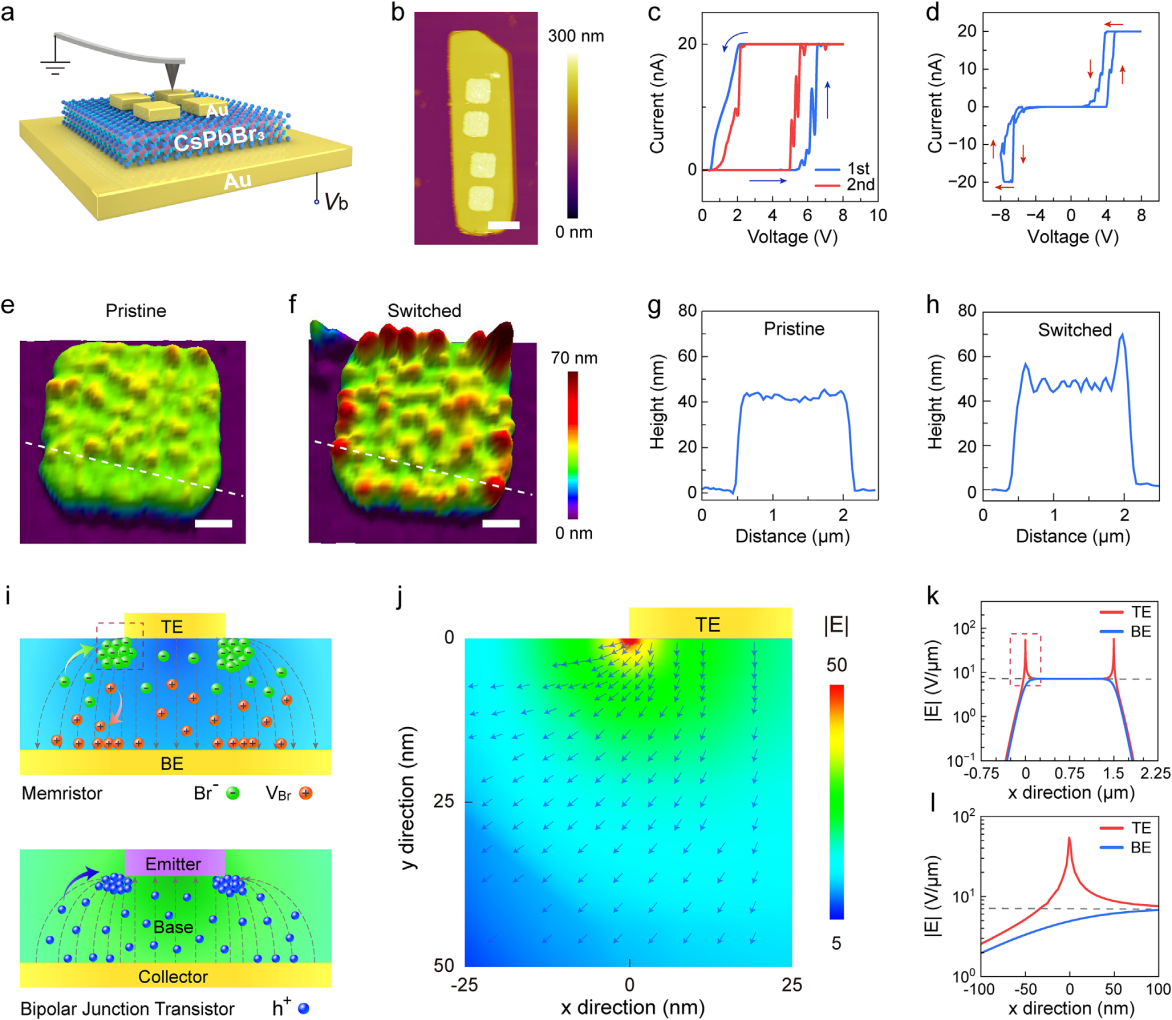
Switching behavior in downscaled devices with TE dimension of 1.5 × 1.5 µm. (a) Schematic drawing of the device structure and c‐AFM setup. (b) Morphology mapping of a NP with four patterned TEs. Scale bar: 2 µm. (c‐d) *I*–*V* curves of a typical device under positive (c) and bidirectional (d) voltage sweeps. The arrows denote the sweeping direction. All tests were conducted in dark. (e‐f) AFM images of a TE before (e) and after (f) voltage sweeps. Scale bars: 300 nm. (g‐h) Corresponding height profiles extracted along the white dashed lines in (e) and (f), respectively. (i) Illustration of cross‐sectional electric field distribution in a unilaterally downsized memristor (top) and a bipolar junction transistor (bottom). (j) TCAD simulation of field intensity near the edge of TE. The simulated area corresponds to the red dashed box in (i). The arrows indicate the direction of field. The unit for |E| is V/ µm. (k) Line‐profiles of field intensity at bottom of TE (red) and top of BE (blue). The x = 0 position refers to the left edge of TE. The gray dashed line represents the field from ideal parallel‐plate scenario. (l) Magnified view of |E| from the dashed box in (k).

Subsequently, the TE was further downsized to 1.5 µm × 1.5 µm. To establish electrical connection, a conductive atomic force microscope (c‐AFM) with Pt‐coated tips was employed for the memristor characterization. During the measurement, the grounded tip was in physical contact with the center of TE, while a bias of *V*_b_ was applied to BE (Figure 2a). In order to protect the tip from Joule heating, the *I*_CC_ was limited to 20 nA. Figure 2b shows the morphology mapping of a typical device, where multiple Au TEs were fabricated onto the same NP for better comparison. The current–voltage (*I*–*V*) curves of a single memristor demonstrate switching at +6 V during the first forward sweep, and the device reinstates to HRS during the backward sweep (Figure 2c), consistent with the unipolar volatile switching behavior. By further sweeping the voltage to the negative regime, an additional switching occurs at ‐6.5 V with similar back‐switching during the backward sweep (Figure 2d). Compared to the 3 µm devices, the set voltage is increased by 6‐fold, indicating that the formation of vacancy filaments is likely hindered in these downsized devices. The shift from bipolar nonvolatile switching to unipolar volatile switching ascribes to the low current compliance of 20 nA, which causes too thin conductive filaments to preserve LRS during reverse voltage sweep. To investigate the size effect, we examined the morphology of TEs before and after multiple switching. Compared to the as‐fabricated electrode surface (Figure 2e), the switched electrode exhibits distinct protrusions mostly along the periphery of TE (Figures 2f; S12). The diameter of the protrusions ranges from approximately 100 to 200 nm with height up to 190 nm (Figure 2g,h). In oxide memristors, similar protrusions have been reported, which are attributed to generation of oxygen bubbles below the metal electrode under high bias [61, 62]. Nevertheless, the oxygen bubbles are randomly distributed rather than concentrate to the edge of TE. The morphology modification of TE indicates a switching‐induced edge effect, which may originate from the configuration of the unilaterally downsized device. While the central region of TE is akin to a parallel‐plate setup with uniform electric field, the edge of TE encompasses the bended electric field lines outside the electrode, leading to a fringe field in the vicinity of TE with enhanced intensity. The charged ions (e.g., bromine anions or vacancies in CsPbBr_3_) drift along the fringe field and accumulate near the edge of TE. In the presence of a strong field toward BE, the excessive number of bromine anions crowd up to the top interface, which causes lattice expansion accounting for the observed bumps, as shown in Figure 2i. Such ion crowding effect in the ionic transport system is analogous to the current crowding effect in electrical transport systems. For instance, in bipolar junction transistors (BJTs) and other similar electrode configurations [63, 64, 65] the carrier density at the edge of TE also shows significant increase, resulting in current crowding and consequent local heating (Figure 2i). To evaluate the crowding effect, we conducted numerical simulations of the local field distribution using the Technology Computer‐Aided Design (TCAD). The simulation employed electrode configurations and material properties consistent with the experimental setup (more details in Supplementary Note S1). Under bias of ‐1 V, the local electric field intensity near the edge of TE demonstrates substantial enhancement (Figure 2j), with an over 2‐fold increase in field intensity localized across lateral size of ∼50 nm (Figures 2k,l; S13). The region with enhanced field is consistent with the dimension of the protrusions. In previous reports, it has been shown that the bromine anions and vacancies in CsPbBr_3_ are mobile under field intensity of ∼1 V/µm [51]. Thus, the enhanced fringe‐field promotes the drift of ions toward the edge of TE, which possibly accounts for the morphology change.

### 2.2 Downscaling Device Footprint to Nanometer Level

**FIGURE 3:**
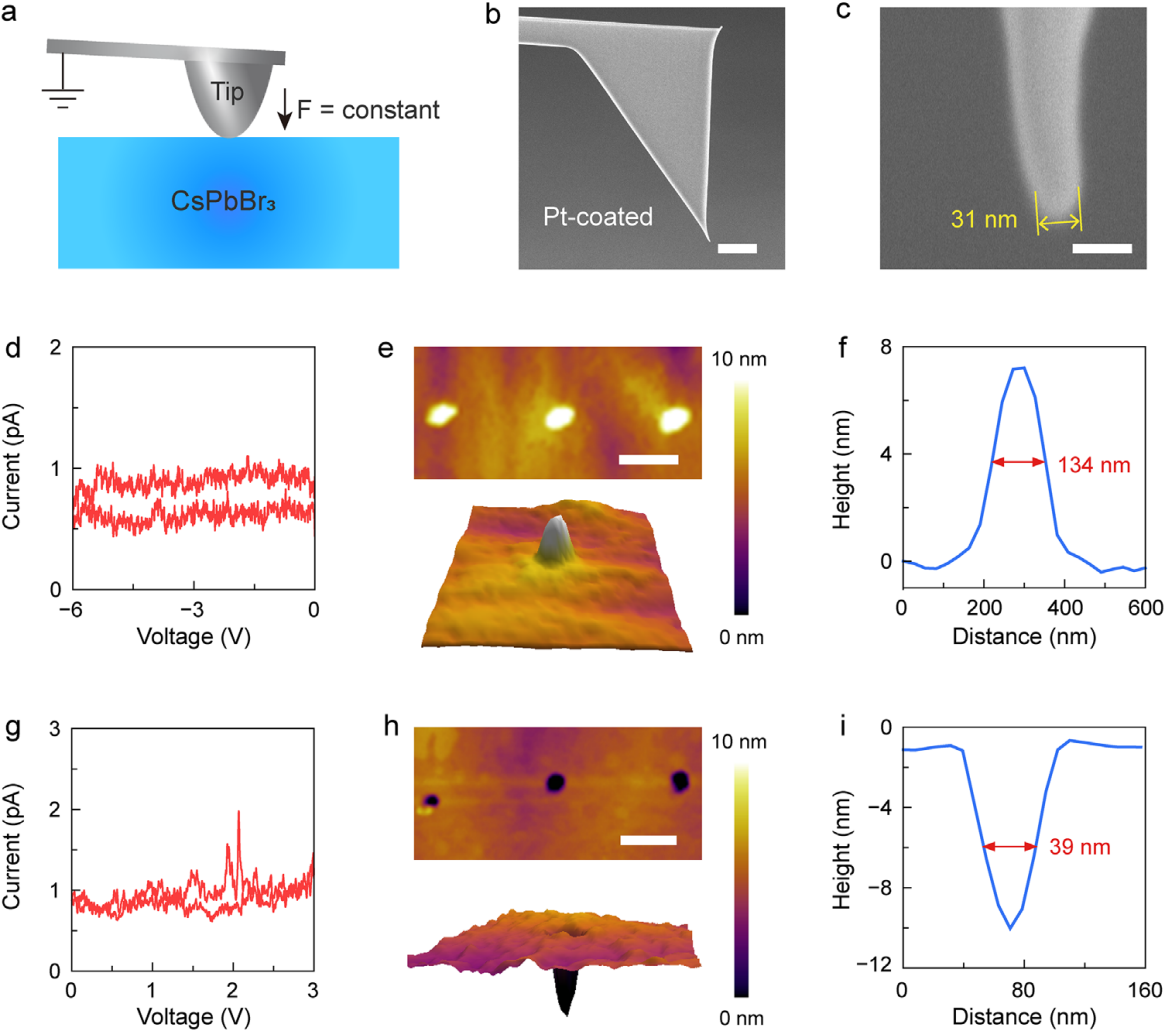
In situ c‐AFM characterizations of the ion crowding effect in a single NP. (a) Schematic illustration of the experimental setup. (b) SEM image of a c‐AFM tip used in memristor measurements. (c) Zoom‐in view of the tip in (b). (d) Local *I*–*V* sweep between 0 and ‐6 V at scanning rate of 0.2 V/s. (e) AFM image (top) of local morphology after negative voltage sweeps at three consecutive locations. The 3D topographical image of a typical protrusion is shown in the bottom image. (f) Height profile of a single protrusion. (g) Local *I*–*V* sweep between 0 and +3 V at scanning rate of 0.2 V/s. (h) Local morphology (top) after positive voltage sweeps at three consecutive locations. Bottom: 3D topographical image of a typical indentation. (i) Height profile of a single indentation. Scale bars: (b) 3 µm, (c) 50 nm, (e) 400 nm, (h) 200 nm.

To confirm the proposed crowding effect, the Pt‐coated AFM tip was directly employed as a TE (Figure 3a). As shown in the side‐view SEM images (Figure 3b,c), the diameter of the conductive tip is approximately 30 nm. During the c‐AFM measurement, the tip maintains in physical contact with the NP under a constant downward force. The magnitude of force was set as small as possible to prevent causing morphology deformation by the tip. To induce the crowding effect, a single voltage sweep was first applied, followed by immediate morphology examination of the local area. Upon a negative voltage sweep between 0 and ‐6 V, the conduction current is almost undetectable (Figure 3d), suggesting that no complete CF was formed. Whereas, the local morphology of the NP shows apparent raise similar to that on the gold TE. Figure 3e demonstrates the AFM mapping after three consecutive voltage sweeps at separate locations. All locations consistently exhibit conical protrusions with height of approximately 7 nm. The diameter of the protrusions is around 130 nm, defined by the full‐width at half‐maximum (FWHM) of the peak in height profile (Figure 3f). Oppositely, under a positive voltage sweep, the local morphology shows a circular indentation at each location with no conduction current measured (Figure 3g,h). Compared to the protrusions, the indentations exhibit similar depth but smaller diameter with average FWHM of 39 nm (Figure 3i), which may be convoluted by the diameter of the AFM tip. The polarity‐dependent formation of local bumps and dents strongly suggests migration and accumulation of ions and vacancies, which lead to expansion or shrinking of the perovskite lattice.

**FIGURE 4:**
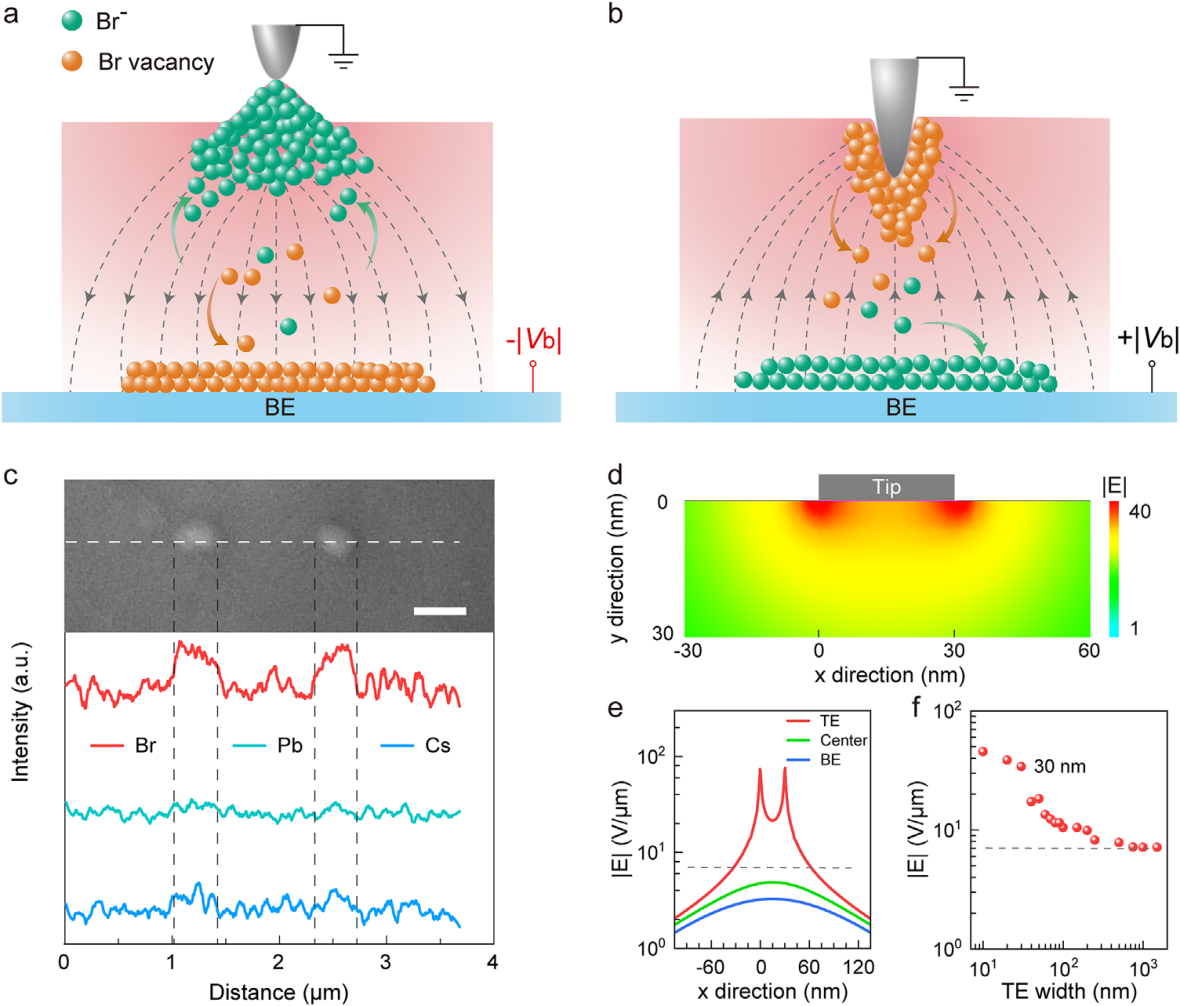
Mechanism and verification of the ion crowding effect. (a‐b) Illustrations of ion transport and distribution under negative (a) and positive (b) bias on the BE. (c) Top: SEM image of two protrusions. Scale bar: 500 nm. Bottom: corresponding EDS line‐scan profiles of Cs, Pb and Br along the white dotted line across the two protrusions. (d) Simulated electric field strength near the tip at bias of ‐1 V. The unit for |E| is V/µm. (e) Line‐profiles of field intensity at top (red), center (green) and bottom (blue) of the NP. The x = 0 position refers to the left edge of TE. The gray dashed line represents the field from ideal parallel‐plate scenario. (f) Simulated |E| at the top middle of the NP as a function of TE width.

In the scenario of a sharp‐tip TE, the size of the tip is comparable to the scope of the ion crowding effect. Consequently, the electric field under the entire TE is enhanced due to overlap of the fringe field. When BE is negatively biased, the mobile bromine anions drift reversely along the electric field and accumulate near the tip (Figure 4a). The strong field in the vicinity generates interstitial bromine anions, which locally expands the volume of lattice. Conversely, upon applying a positive bias to BE (Figure 4b), the bromine vacancies drift along the field and accumulate at TE. Under high local field, the bromine vacancies may concentrate to the vicinity of TE, resulting in local structural collapse and corresponding dents. Notably, the size of the bumps is apparently larger than that of the dents. This is due to the fact that more vacancies can be concentrated into a fixed volume than interstitial bromine anions. Such asymmetric behavior between interstitial bromines and vacancies has also been observed in CsPbBr_3_ nanowires [66], where the vacancies are more prone to accumulation than the anions under a moderate electric field. In order to further verify the above mechanism, we analyzed the composition of the surface bumps with EDS characterization. As shown in Figure 4c, the elemental distribution demonstrates apparent aggregation of bromine in the bumps, while the content of cesium and lead remains unchanged. This direct observation of local bromine enrichment confirms the crowding effect in the system. Moreover, we also conducted numerical simulations to examine the strength of local field. As illustrated in the distribution of field intensity (Figure 4d), the local electric field surrounding the entire tip is enhanced from overlap of the fringe field. The field intensity near TE exhibits up to 6‐fold increase compared to that at the NP center and BE (Figure 4e). Notably, the field below the center of the tip also greatly exceeds the parallel‐plate capacitor limit. These results suggest that the crowding effect becomes dominating at nanoscale. By tracking the field intensity below the center of the tip as a function of TE width, it is shown that the superimposition of the fringe field occurs as the CD of TE falls below a few hundred nanometers, and is further intensified in the sub‐50 nm regime (Figure 4f). Therefore, the crowding effect should be taken into consideration in sub‐micrometer memristor design. Practically, the net electric field is likely weaker than the simulation value, owing to an internal counter‐field from accumulation of the ions.

### 2.3 Dynamics of Crowding Effect and Influence on Resistive Switching

**FIGURE 5:**
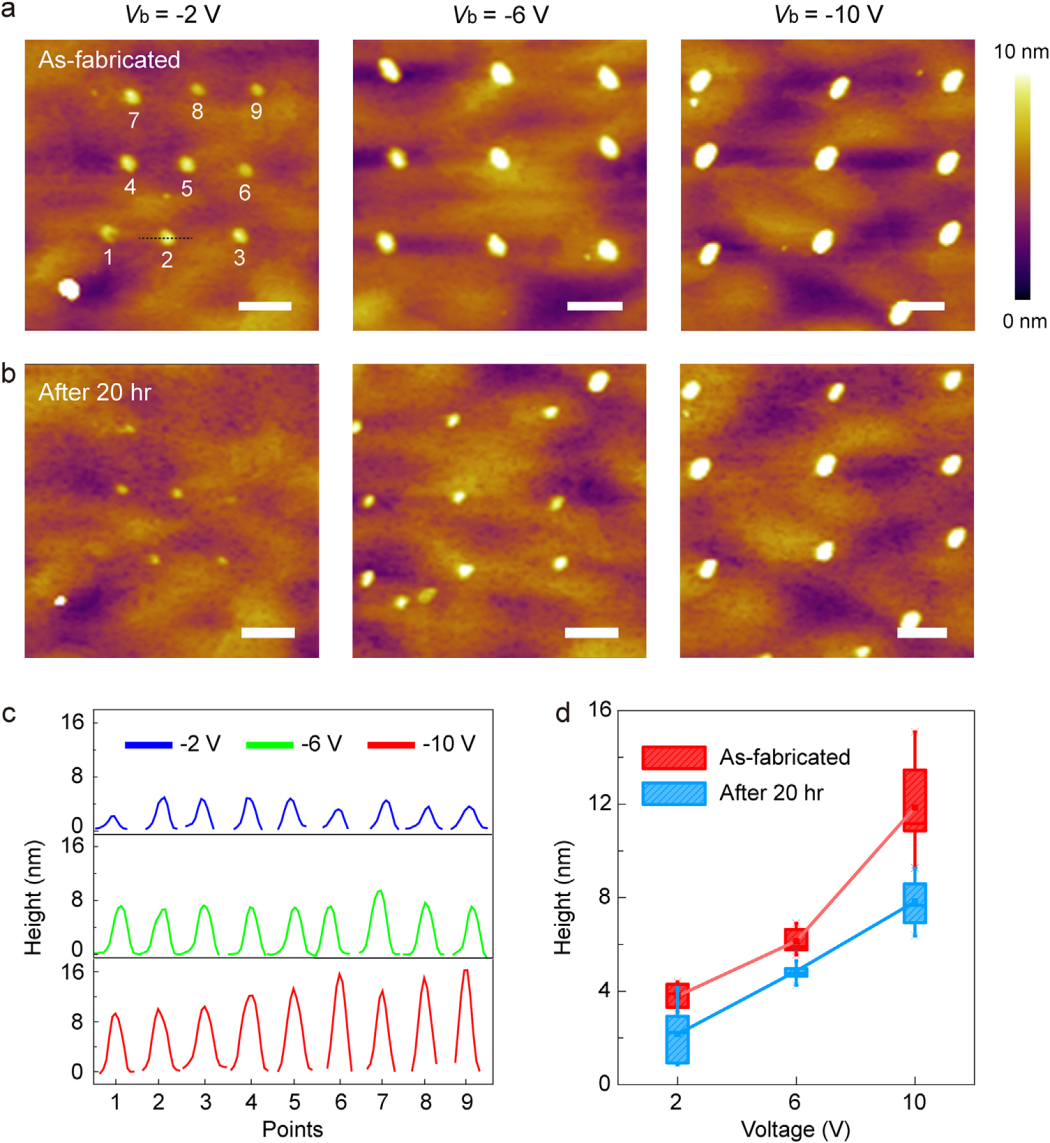
Field‐dependency and evolution of the protrusions. (a) AFM images of 3 × 3 protrusion arrays fabricated by voltage sweeps to ‐2 (left), ‐6 (middle) and ‐10 V (right). The scanning rate is 0.2 V/s. The numbers in the left image denote the fabrication sequence. (b) Morphology of (a) after storage for 20 h in a N_2_‐filled glove box. All scale bars are 500 nm. (c) Local height profiles at each protrusion right after the voltage scan. (d) Statistical distribution of protrusion height in the arrays upon fabrication (red) and after storage in N_2_ for 20 h (blue).

To gain more insights into the crowding effect, we further investigated the evolution of field‐induced protrusions under various bias voltages. Multiple arrays of protrusions were fabricated on the same NP by varying the magnitude of the stimulation voltage. Each array contains 3 × 3 bumps formed under the same condition. No conduction current was observed during the fabrication, eliminating the influence from Joule heating. The local morphology was then captured immediately upon fabrication and after storage for 20 h inside a nitrogen‐filled glove box. As shown in Figures 5a and S14 (Supporting Information), the height and size of individual protrusions are consistent with each other in a single array, and demonstrate evident correlation to the applied voltage. The raise of the surface is proportional to the bias, and the average height of the bumps at ‐2, ‐6, and ‐10 V features respectively 3.6, 7.2, and 10.9 nm, as measured from the cross‐section of the height profiles at individual locations (Figure 5c). Additionally, similar study was also performed under positive bias, in which arrays of uniform dents were created (Figure S15). After storage for 20 h, all protrusions exhibit reduced height compared to their as‐fabricated states (Figure 5b). Particularly, the ‐2 V induced bumps are nearly restored, while those stimulated at higher voltages are only partially reversed (Figure 5d). Further long‐term observation of the surface suggests that the bumps formed under ‐10 V remained even after ten days (Figure S16). The decay of the ion crowding effect is consistent with the dynamics of bromine anions in monocrystalline CsPbBr_3_. Under moderate electric field, the ion migration is spontaneously reversible [56], presumably driven by the concentration gradient of ions and internal electric field. Nevertheless, under overly high field, the deformation of surface becomes permanent due to large number of interstitial sites inside the crystal or loss of bromine outside the lattice. The highly controllable formation of uniform arrays on the NP surface suggests that the crowding effect is tunable down to sub‐50 nm.

**FIGURE 6:**
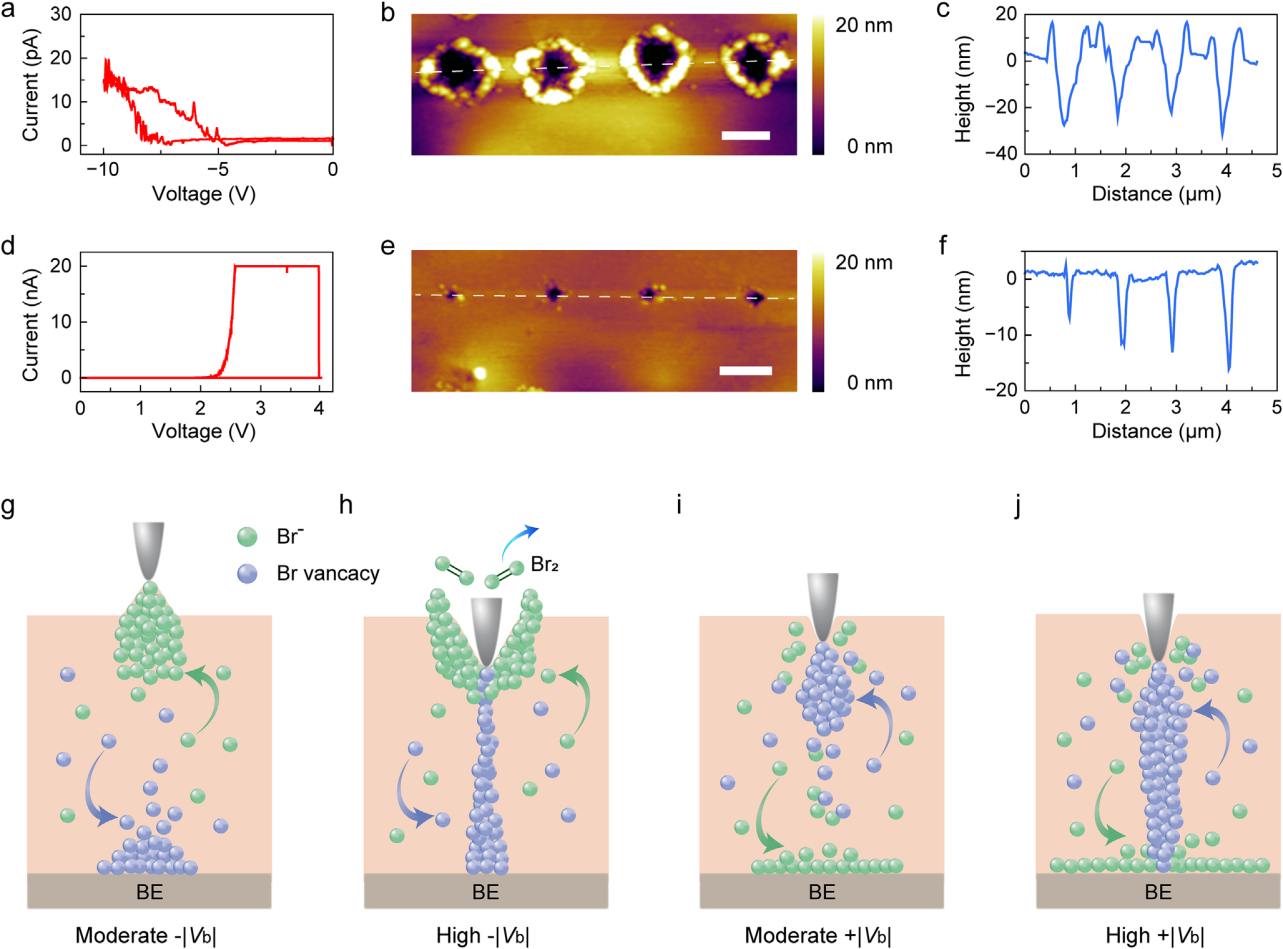
Electrical switching and surface deformation of the nanometer sized devices. (a) Local *I*–*V* characteristics after a negative voltage sweep to ‐10 V. The scanning rate is 0.2 V/s. (b) Topographic image of four consecutive locations upon switching. (c) The corresponding height profile along the white dashed line in (b). (d) Local *I*–*V* curve after a positive voltage sweep to 4 V. (e) Topographic image of four consecutive locations upon switching. (f) The corresponding height profile along the white dashed line in (e). All scale bars are 500 nm. (g‐j) Schematic illustrations of CF formation in negatively (g‐h) and positively (i‐j) biased devices.

Owing to the crowding effect, the accumulated anions near the anode of device hinder the formation of vacancy CFs, resulting in greatly increased threshold for resistive switching in downsized devices. Nevertheless, occasionally in thinner NPs, we were able to achieve switching by sweeping to a high voltage. As shown in the *I*–*V* curve (Figure 6a), upon sweeping to ‐10 V, the nano‐device gradually transits to LRS with current increasing to ∼20 pA, and reinstates to HRS during the backward sweep. The morphology of the switched device was subsequently examined. Interestingly, the four sequential voltage scans at separate locations consistently create the same complex structure, where a central indentation is surrounded by outside protrusion (Figure 6b). The cross‐section of the “hole‐in‐a‐bump” topography exhibits average lateral size of 770 nm with a central concavity (Figure 6c). The average depth of the indentation is 24.3 nm with outside protrusion of 15.5 nm. Comparatively, the electroforming process in positively biased devices is more facile. The device abruptly switches to LRS at around +4 V, and returns to HRS during the backward sweep (Figure 6d). The LRS current reaches the compliance limit of 20 nA, indicating substantially increased size of CFs. Curiously, the surface of the switched devices shows no significant modification even with an over 10^4^ increase of current magnitude (Figure 6e). Compared to that formed at lower bias, the average lateral size of the dents features only a slight increase to 95 nm with depth of 10∼20 nm (Figure 6f). These unique switching behaviors are also a direct result of the ion crowding effect. As shown in Figure 6g, when negatively biased, the bromine anions accumulate near the AFM tip to form a protrusion, while bromine vacancies drift to the BE. As bias increases, a vacancy filament grows upward from the BE. However, the filament is difficult to cut through the bromine‐rich protrusion and forms a complete conductive pathway, owing to the constant recombination between interstitial anions and vacancies. Under sufficiently high field, the extra anions are oxidized into free bromine, leaving a large dent in the center of a protrusion. The central dent allows electrical connect between the tip and filament, resulting in switching and the observed “hole‐in‐a‐bump” topography (Figure 6h). On the contrary, in positively biased devices, the vacancies accumulate near the tip, leading to an indentation comparable to the size of the tip (Figure 6i). Upon increasing the bias, the vacancy filament directly grows from the indentation. As the large BE is almost unaffected by the crowding effect, the formation of filament requires little competition with the ion‐vacancy recombination (Figure 6j). Therefore, a stronger filament can be formed, leading to the observed high conduction current and low switching threshold. These results suggest that the ion crowding effect can become dominating in nanometer‐sized memristors with severe local surface deformation, while properly applied bias might minimize the deformation and survive from the crowding effect. In addition, further simulation of crossbar arrays shows that the crowding effect becomes bilateral in such architecture and affects both TE and BE, which may add difficulty to the downscaling of devices (Figures S17 and S18). The ion migration and vacancy redistribution are more intense at the electrode edge, which may facilitate the formation of CFs. Especially in the case of active metal electrodes, the role of the ion crowding effect is worth looking forward to. The ion crowding effect provide new insight for bromine‐vacancy conductive mechanism systems, are required to guide further miniaturization of memristors. Moreover, in hybrid halide perovskites, both the cations and anions are mobile under intense electric field [67], further complicating the ion crowding phenomenon. In addition, the enhancement of electric field can also exist in other downsized devices such as oxide memristors. Nevertheless, the less mobile oxygen anions and vacancies would require a significantly larger electric field, which tends to reduce the anions to oxygen gas rather than form protrusions and indentations [62]. Furthermore, the insufficient population of mobile ions in oxides may result in undetectable crowding effect. In other soft lattice systems such as certain organic crystals or metal‐organic frameworks (MOFs), the similar crowding effect may also be present but has yet to be demonstrated. Overall, the crowding effect uniquely found in soft lattice systems provides deepened understanding of ionic behavior under strong electric field at micro‐ and nanoscale, facilitating the development of ultraminimized perovskite memristor arrays.

## 3 Conclusion

In summary, we show that the downsizing of perovskite‐based memristors can be governed by the ion crowding effect, particularly when the device footprint is comparable to or below micrometer level. The crowding effect originates from substantially enhanced fringe field near the edge of TE, which concentrates mobile anions or vacancies and forms respective local protrusion or indentation. The corresponding resistive switching in these micro‐ and nanodevices exhibits distinct features including increased threshold and unique morphology. Such ion crowding effect is expected to broadly exist in halide perovskites with soft lattice and mobile ions. In addition, the well‐controlled ionic behavior at micro‐ and nanoscale provides insight into designing and fabrication of arrayed memristive devices with reduced footprint.

## 4 Experimental Section

### 4.1 Synthesis of CsPbBr_3_ Single Crystal NPs

CsPbBr_3_ NPs were synthesized via a modified antisolvent recrystallization process. CsBr (99.999%, Sigma‐Aldrich) and PbBr_2_ (99.999%, Sigma‐Aldrich) powders with a 1:1 molar ratio were dissolved in N,N‐dimethylformamide (DMF), and vigorously stirred for 30 min. The precursor solution was filtered by a nylon 66 filter head with 0.22 µm porous. Then, 10 µL precursor solution was dropped on a cleaned FTO glass, and placed by a simple support frame in breaker with a small amount of isopropanol. After 8 h, yellow crystals formed on the FTO substrate. The substrate was rinsed in isopropanol and dried on a heated plate at 80 °C for 2 min.

### 4.2 Device Fabrication

For basic microscale memristor, the BEs (15 nm Ti and 35 nm Au) were fabricated on a SiO_2_/Si substrate by photolithography and e‐beam evaporation, followed by a lift‐off process. The as‐grown NPs were transferred onto Au BEs via a micromanipulator. The TEs (240 nm Ag and 20 nm Au) or 60 nm Au were subsequently patterned by the electron‐beam lithography method, followed by evaporation and lift‐off processes. The CsPbBr_3_ NP was well‐preserved throughout the entire fabrication processes, as confirmed by OM and SEM characterizations (Figure S5). For nanosized device, ITO substrates were treated with UV‐ozone illumination for 30 min. Subsequently, the as‐grown NPs were transferred onto ITO using a micromanipulator.

### 4.3 Sample Characterization

SEM and EDS line scanning were performed using a JSM‐7900F field‐emission SEM instrument equipped with an Oxford X‐MAX energy dispersive X‐ray spectroscope. XRD pattern was acquired by a Smart Lab 9 KW (Nippon Neotoku Corporation) with Cu Kα radiation under an operating voltage of 240 kV. PL images and spectra were collected using an Olympus BX53F wide field microscope equipped with a spectrometer (Princeton Instrument, SpectraPro, HRS‐500s) under excitation from a mercury lamp.

### 4.4 Electrical and c‐AFM Measurements

The memristor performance was measured using a probe station equipped with Keithley 2450 Sourcemeter (*I*–*V* characteristics) and the Keithley 4200A‐SCS Parameter Analyzer (pulse measurements). The DC scan step is 0.01 V for Au‐based device and 0.04 V for Ag‐based device. The in situ c‐AFM measurements were carried out using a multimode AFM system (MFP‐3D origin+, Asylum Research). Pt‐Ir‐coated conductive probes (AC240, TM) with a resonant frequency of 70 kHz and a spring constant of 2 N/m were used in the measurements. All topographic images were obtained in tapping mode.

## Conflicts of Interest

The authors declare no conflicts of interest.

## Supporting information




**Supporting File**: advs74241‐sup‐0001‐SuppMat.docx

## Data Availability

The data that support the findings of this study are available from the corresponding author upon reasonable request.
