# Supplementary material for: Ion Crowding Effect in Unilaterally Downsized Perovskite Memristors
Source: Adv Sci (Weinh). 2026 Feb 4;13(21):e24258. doi: 10.1002/advs.202524258 (PMC13073325; doi:10.1002/advs.202524258)
Supplement: Supplementary file 1 — Supporting File: advs74241‐sup‐0001‐SuppMat.docx [file ADVS-13-e24258-s001.docx]

**Supporting Information**

**Ion Crowding Effect in** **Unilaterally Downsized Perovskite Memristors**

*Conghui Tan^1,#^, Meiqi An^2,#^, Weili Liu^1,#^, Haotian Wang^1,#^, Shuai Yang^1^, Jing Li^1,3^, Jianliang Li^1^, Yingmin Luo^1^, Chao Wang^4^, Jiao Xu^1,*^, Yaodan Chi^4,*^, and Yiming Yang^1,*^*

^1^School of Integrated Circuits, Dalian University of Technology, Dalian 116620, China

^2^School of Instrument and Electronics, North University of China, Taiyuan 030051, China

^3^Key Laboratory of Materials Modification by Laser, Ion, and Electron Beams (Ministry of Education), School of Physics, Dalian University of Technology, Dalian 116024, China

^4^Key Laboratory of Architectural Cold Climate Energy Management, Ministry of Education, Jilin Jianzhu University, Changchun 130118, China

*Corresponding author,

e-mails: xujiao@dlut.edu.cn; chiyaodan@jlju.edu.cn; ymyang@dlut.edu.cn

#These authors contributed equally to this work.

**Table S1. Comparison of on/off ratio and electrode dimensions among various perovskite memristors.**

| Device structure | Electrode dimensions (µm) | On/off ratio | Ref |
| --- | --- | --- | --- |
| Au/CsPbBr_3_/Au | 3 × 3 | 10^7^ | This work |
| Ag/CsPbBr_3_/Au | 3 × 3 | 10^8^ | This work |
| Ag/CsPbBr_3_/Ag | 100 × 100 | 10^9^ | ^[1]^ |
| Au/MAPbI_3_/Au | 50 × 50 | 10^8^ | ^[2]^ |
| Ag/PMMA/CsPbI_3_/Pt | 50 × 50 | 10^7^ | ^[3]^ |
| Ag/BA_2_MA_n−1_Pb_n_I_3n+1_/Pt | π × 50 × 50 | 10^7^ | ^[4]^ |
| Ag/RP-V-HP/Pt | 50 × 50 | 10^7^ | ^[5]^ |
| EGaIn/MAPbI_3_/PEDOT:PSS/ITO | π × 20 × 20 | 4.3 × 10^3^ | ^[6]^ |
| Au/Ag/Cs_3_Sb_2_I_9_/ITO | 50 × 50 | 10^5^ | ^[7]^ |
| Ag/Cs_3_Bi_2_Br_9_/ ITO | π × 50 × 50 | 10^8^ | ^[8]^ |
| Al/Cs_3_Bi_2_I_9_/ITO | π × 50 × 50 | 10^4^ | ^[9]^ |
| Ag/(PEA)_2_PbI_4_/ITO | 2000 × 2000 | 4 × 10^4^ | ^[10]^ |
| Al/Cs_3_Bi_2_Br_9_ QD/ITO | π × 50 × 50 | 10^5^ | ^[11]^ |
| Al/Cs_3_Bi_2_I_6_Cl_3_/ITO | π × 50 × 50 | 10^3^ | ^[12]^ |
| Al/MAPbI_3_:3D-SF/ITO | π × 150 × 150 | 10^5^ | ^[13]^ |
| Ag/PMMA/MAPbI_3_/Au | 50 × 50 | 5.26 × 10^5^ | ^[14]^ |

**Supplementary Note 1. Details of TCAD simulation.**

Table S2 provides the key parameters for simulation of the sandwich-structured memristor based on CsPbBr_3_ NPs. The material parameters of CsPbBr_3_ were manually input into the Sentaurus TCAD software. The boundary conditions of both TE and BE were set as ideally ohmic contacts. For the microscale memristors, the dimensions of TE and BE were respectively set as 1.5 × 1.5 μm and 50 × 50 μm. An automatic mesh size was employed, in which the maximum and minimum mesh sizes are 10 nm and 0.01 nm, respectively. For the nanoscale memristors, the dimensions of TE and BE were respectively set as 30 × 30 nm and 3 × 3 μm. The maximum mesh size is 4 nm, and the minimum mesh size is 0.01 nm.

**Table S2. Material parameters of CsPbBr_3_ in the TCAD simulation.**^[15-16]^

| Material parameters | CsPbBr_3_ |
| --- | --- |
| Thickness (nm) | 140 |
| Band gap (eV) | 2.37 |
| Relative permittivity | 16.46 |
| Electron affinity (eV) | 3.5 |
| CB effective density of states (cm^-3^) | 1.75×10^18^ |
| VB effective density of states (cm^-3^) | 1.77×10^18^ |


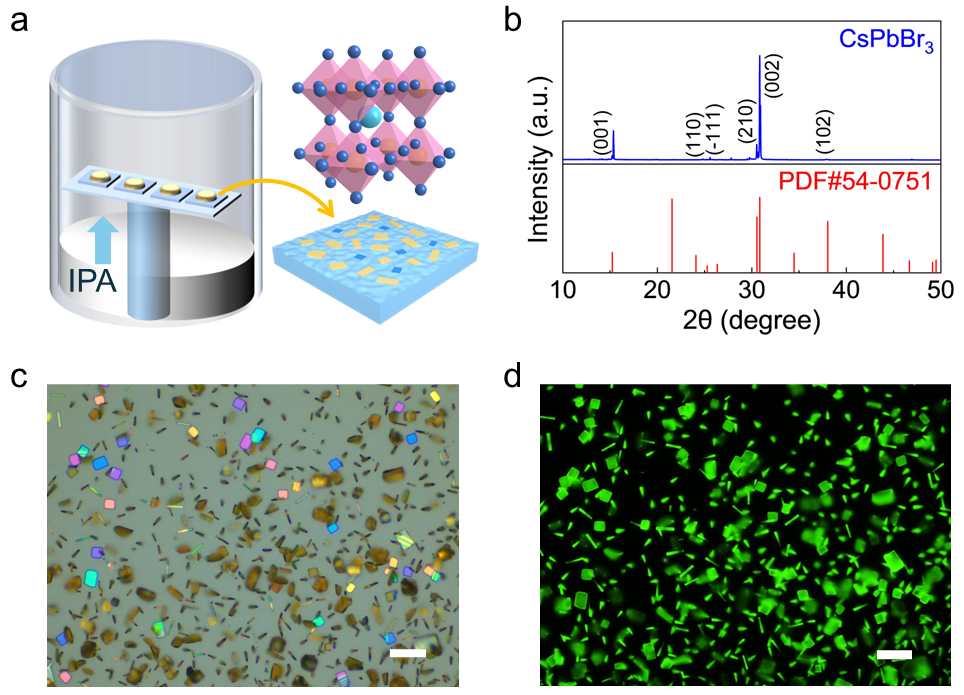


**Figure S1. (a)** Schematic drawing of the synthesis setup. **(b)** The upper panel shows the XRD pattern of the CsPbBr3 NPs. The bottom panel shows the standard XRD pattern of the orthorhombic phase CsPbBr3. **(c)** Top-view optical microscope image of the NPs on a FTO substrate. **(d)** Top-view micro-PL image of the NPs. All the scale bars are 10 µm.





**Figure S2.** AFM images and corresponding height profiles along the black dotted lines of CsPbBr_3_ NPs with the thickness of **(a)** 132 nm, **(b)** 140 nm, **(c)** 147 nm, **(d)** 157 nm, **(e)** 177 nm, **(f)** 260 nm. Scale bars: 2 μm.


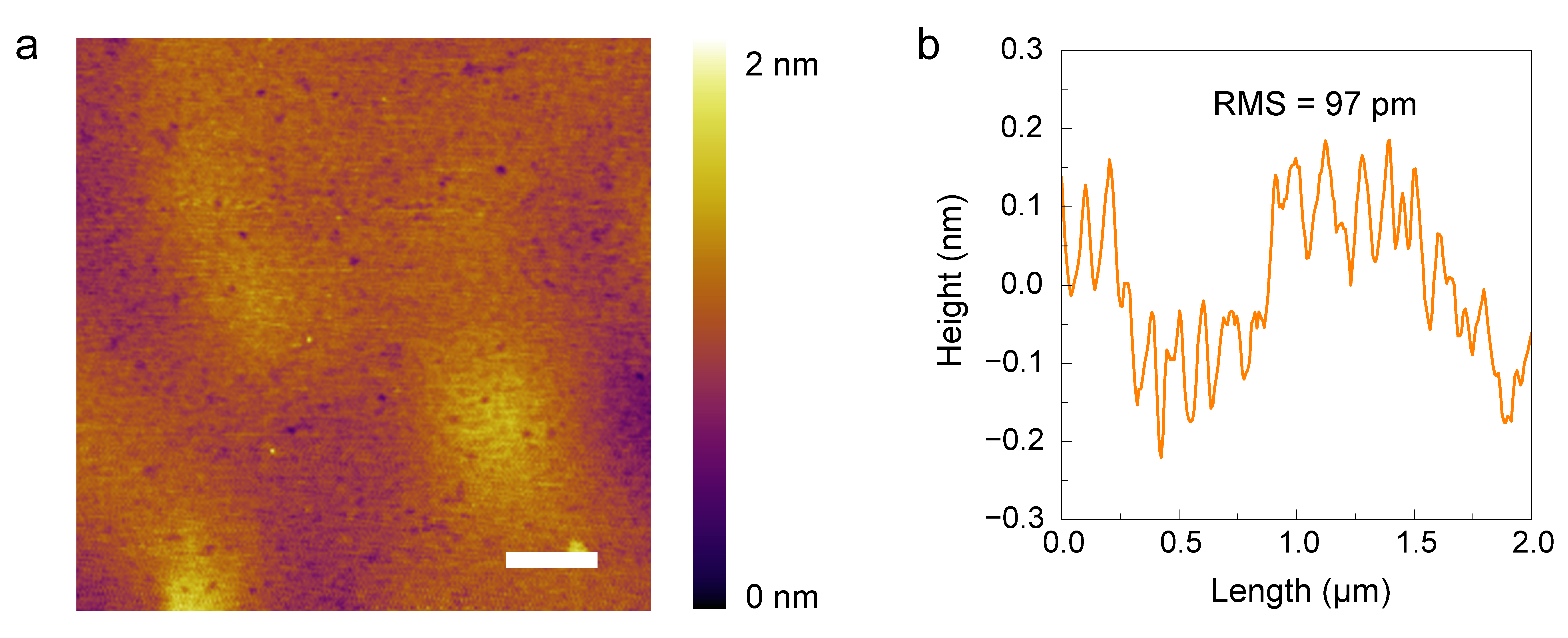


**Figure S3.** **(a)** Local AFM mapping on a CsPbBr_3_ NP. Scale bar: 200 nm. **(b)** Height profile and surface roughness of the NP.


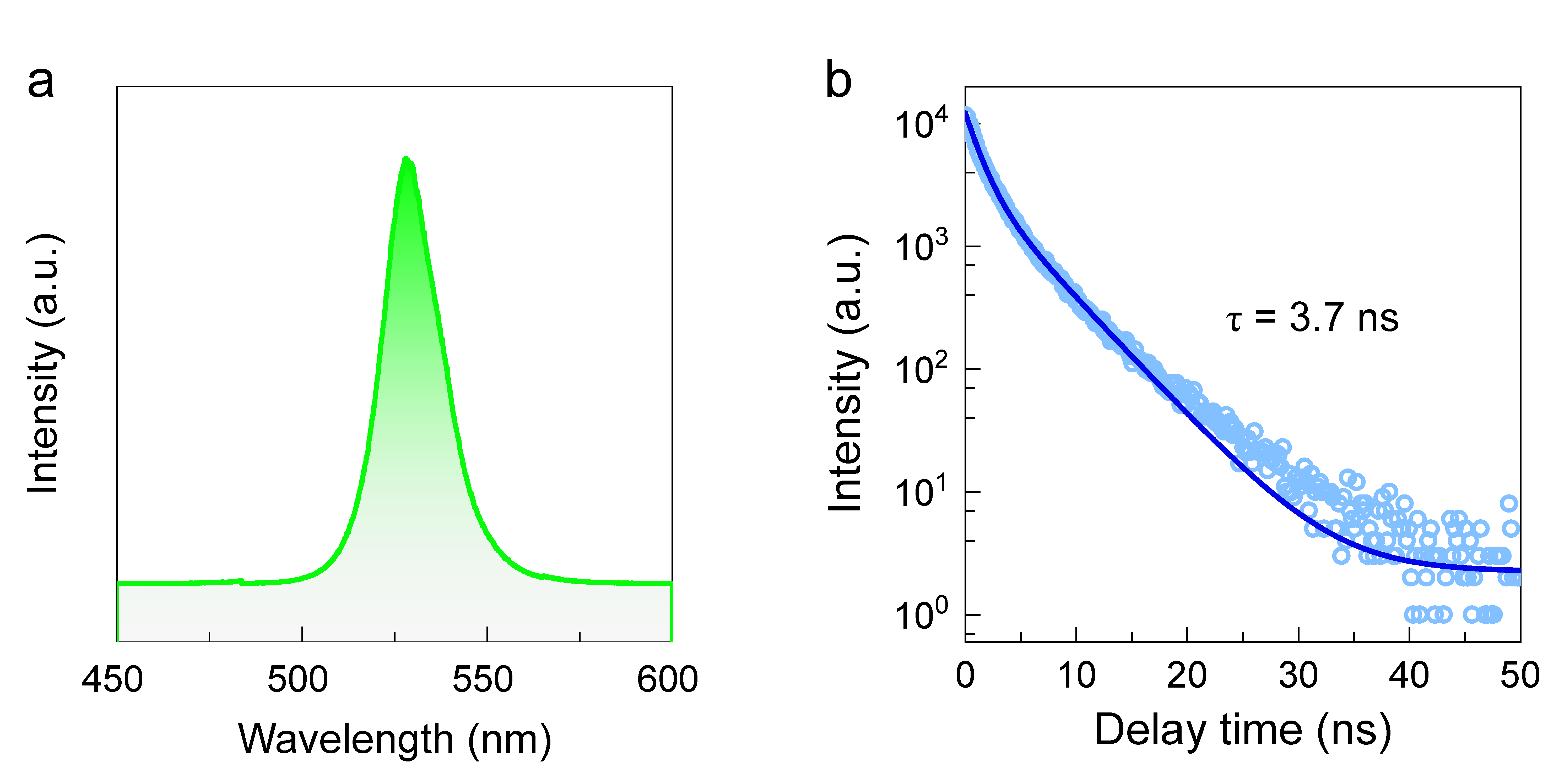


**Figure S4.** **(a)** PL spectrum of a single CsPbBr_3_ NP. **(b)** Time-resolved PL measurement of a typical NP. The blue curve shows the bi-exponential fitting.

The average PL lifetime (τ) of a single CsPbBr_3_ NP was calculated by a bi-exponential fitting of the PL decay.^[17]^

$$\begin{aligned} \text{τ}\text{ }\text{=}\frac{\text{A}\text{1}\tau\text{1}\tau\text{1}\text{+A}\text{2}\tau\text{2}\tau\text{2}}{\text{A}\text{1}\tau\text{1}\text{+A}\text{2}\tau\text{2}}\#\text{(1)} \end{aligned}$$

where A_1_=92.76% and A_2_=7.24% are fitting factors. The fast decay time τ_1_ (2.7 ns) and slow component τ_2_ (7.8 ns) might be respectively attributed to the defect trapping and bulk recombination.^[18]^


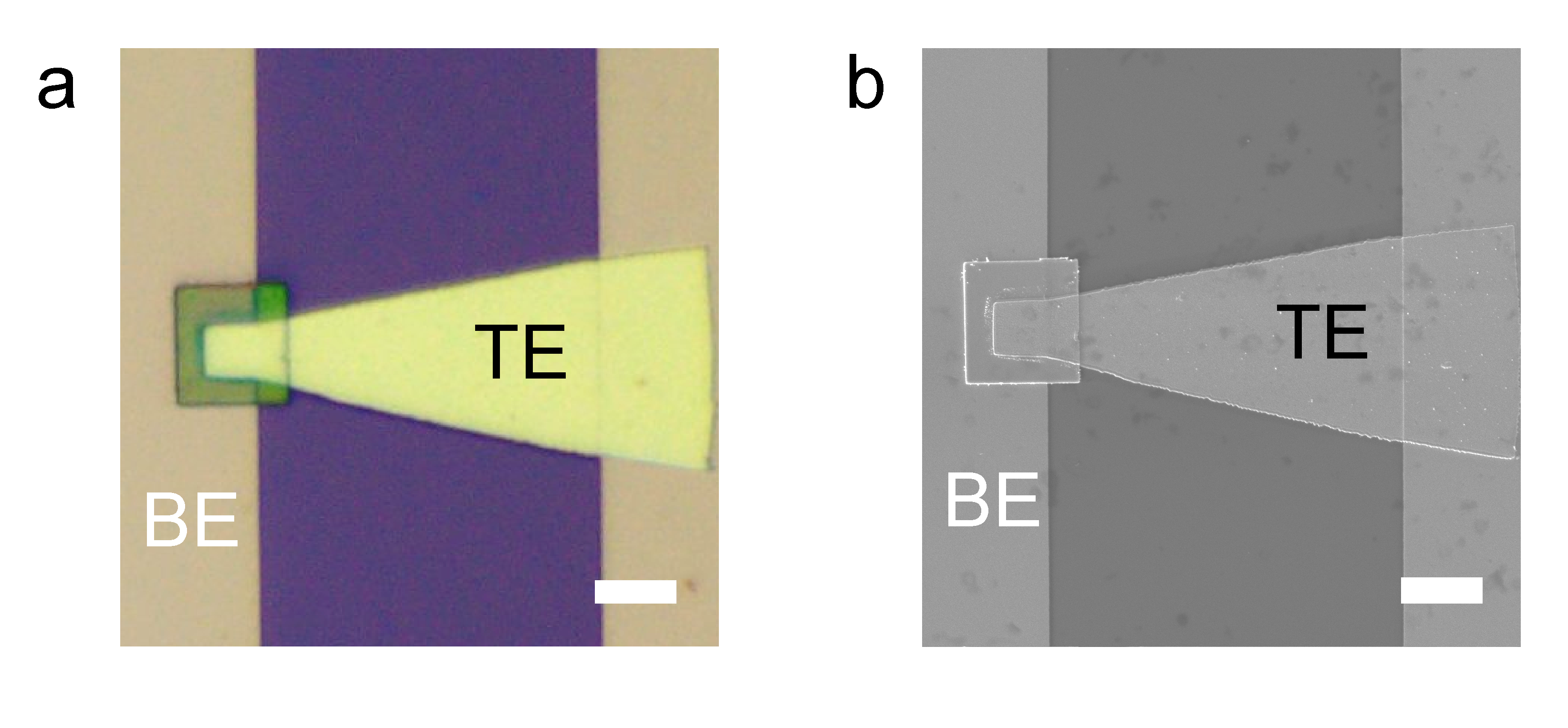


**Figure S5. (a)** Optical microscope image of a memristor device with the top electrode dimension of 3 μm. **(b)** Corresponding SEM image. Scale bars: 5 μm.


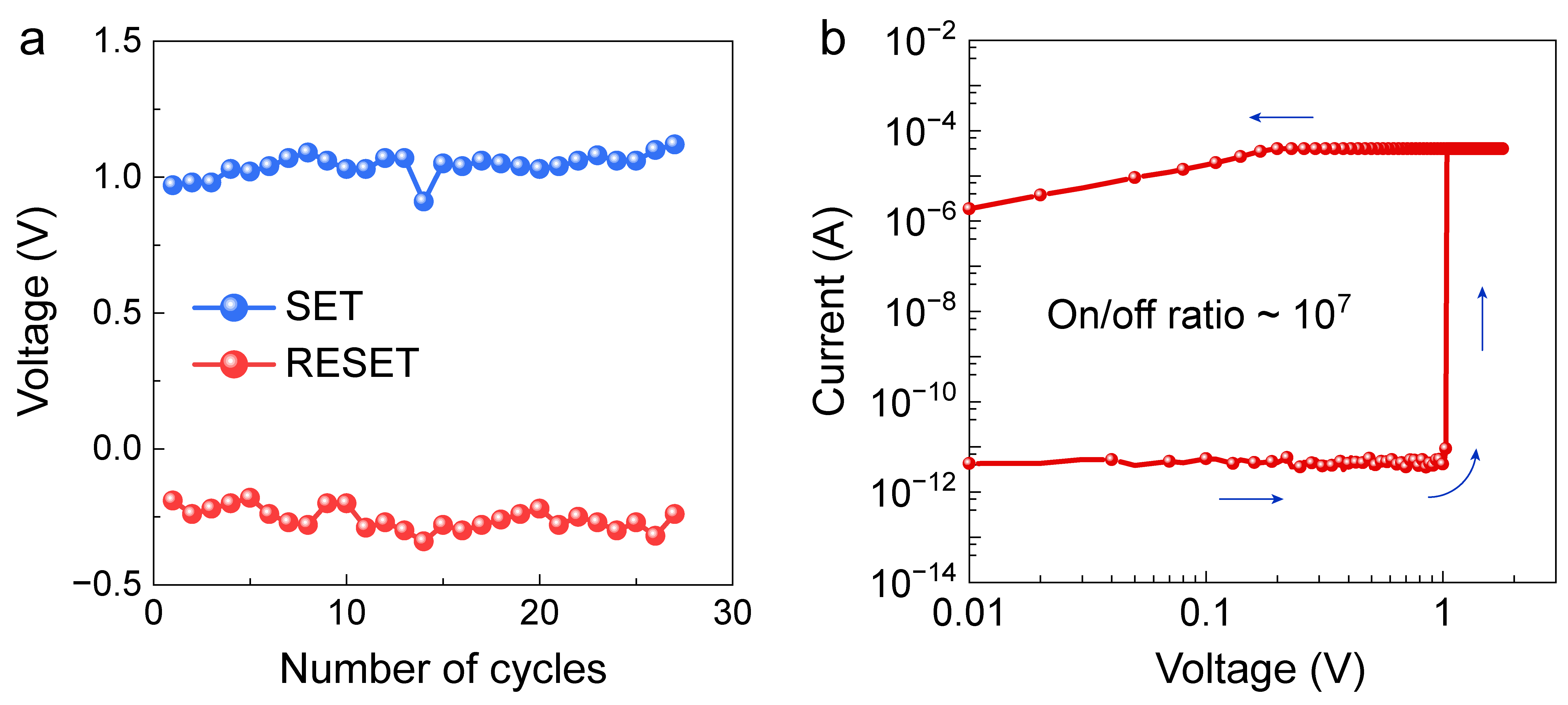


**Figure S6. (a)** Set/reset voltage distribution of the Au/CsPbBr_3_/Au device. **(b)** Double logarithmic scale plot of the *I–V* characteristics for the device.


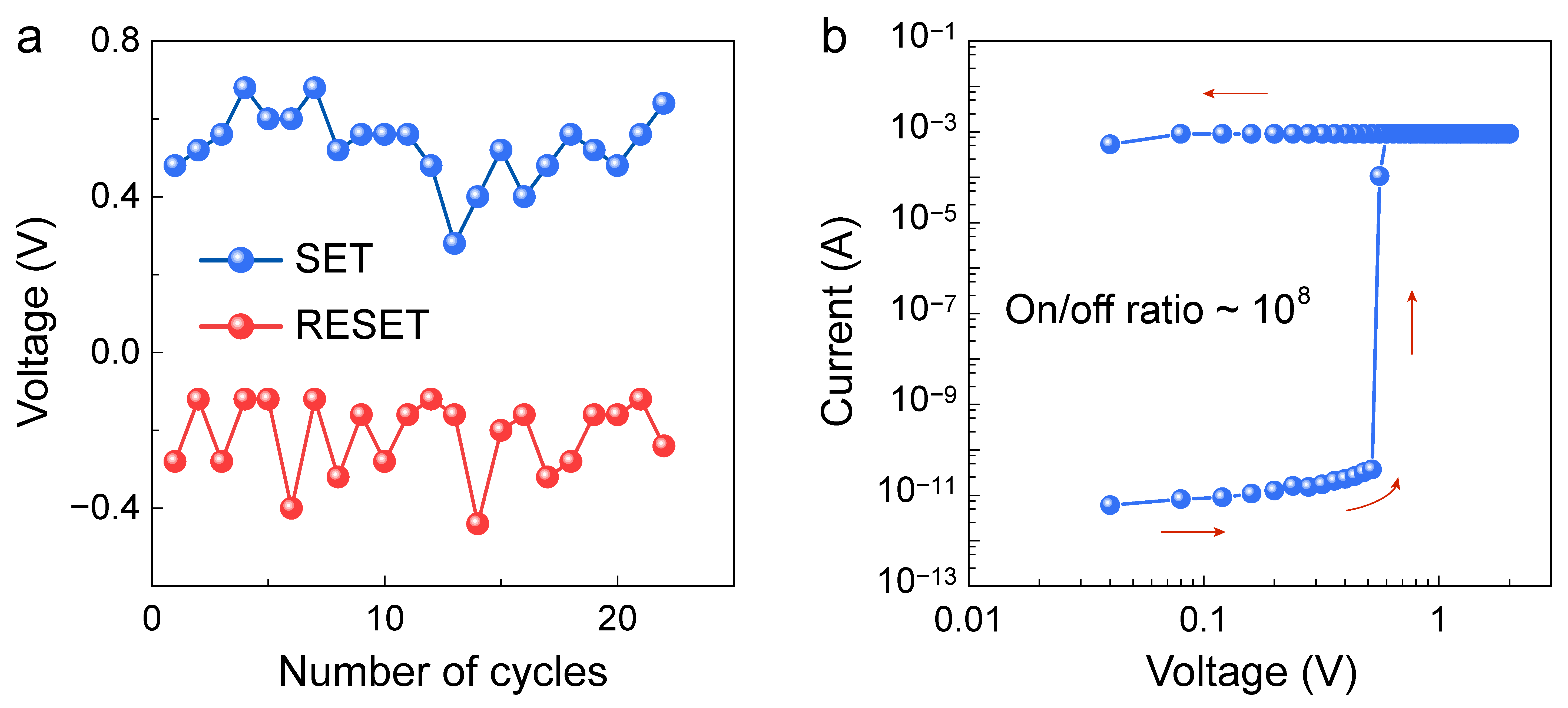


**Figure S7.** **(a)** Set/reset voltage distribution of the Ag/CsPbBr_3_/Au device. **(b)** Double logarithmic plot of *I–V* characteristics for the device.


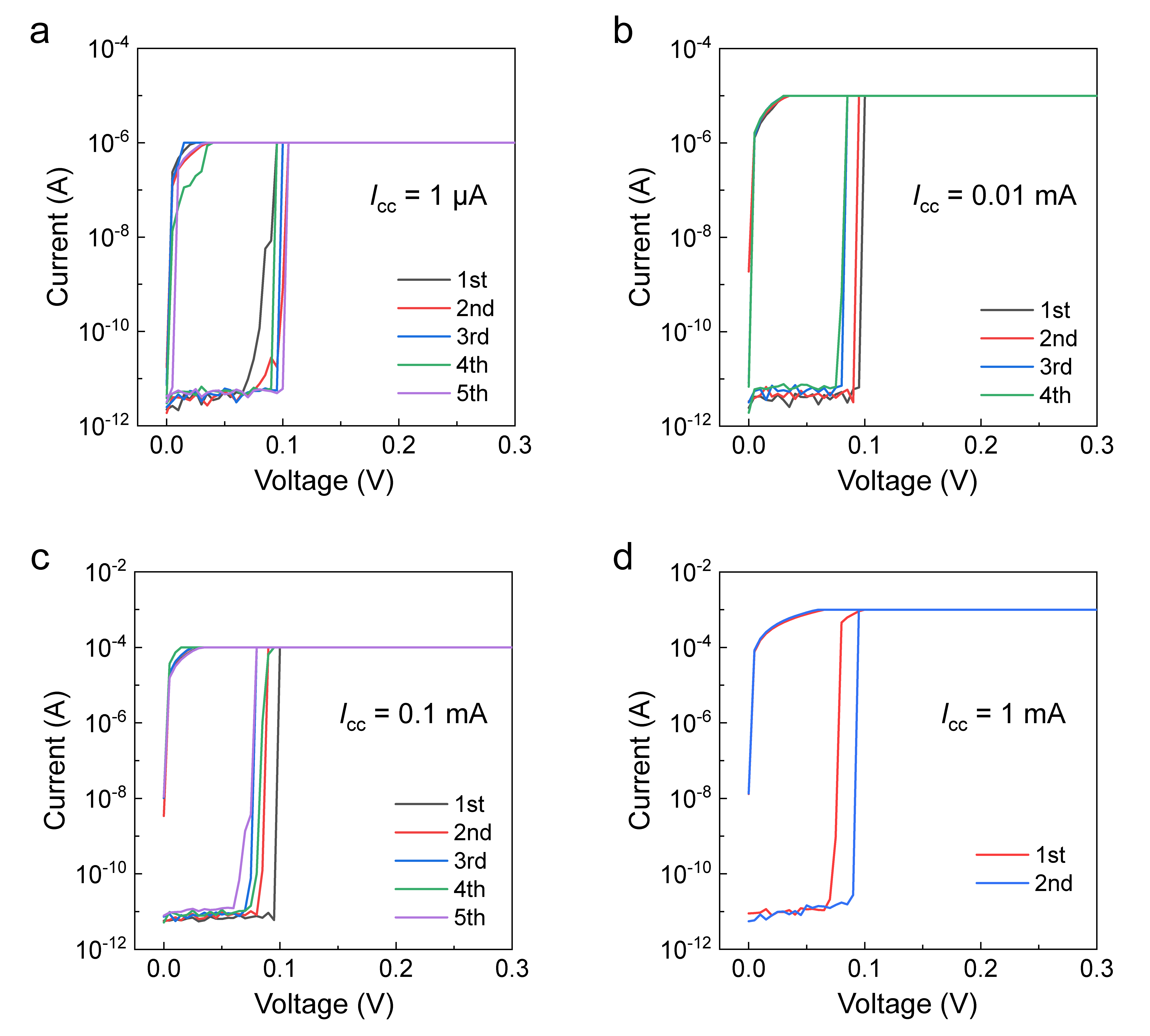


**Figure S8.** Typical volatile threshold switching behaviors of Ag/CsPbBr_3_/Au memristors with *I*_CC_ of **(a)** 1 µA, **(b)** 0.01mA, **(c)** 0.1 mA, **(d)** 1 mA.





**Figure S9.** Typical volatile threshold switching of Au/CsPbBr_3_ devices under *I*_CC_ of (a) 10 μA and (b) 1 μA.


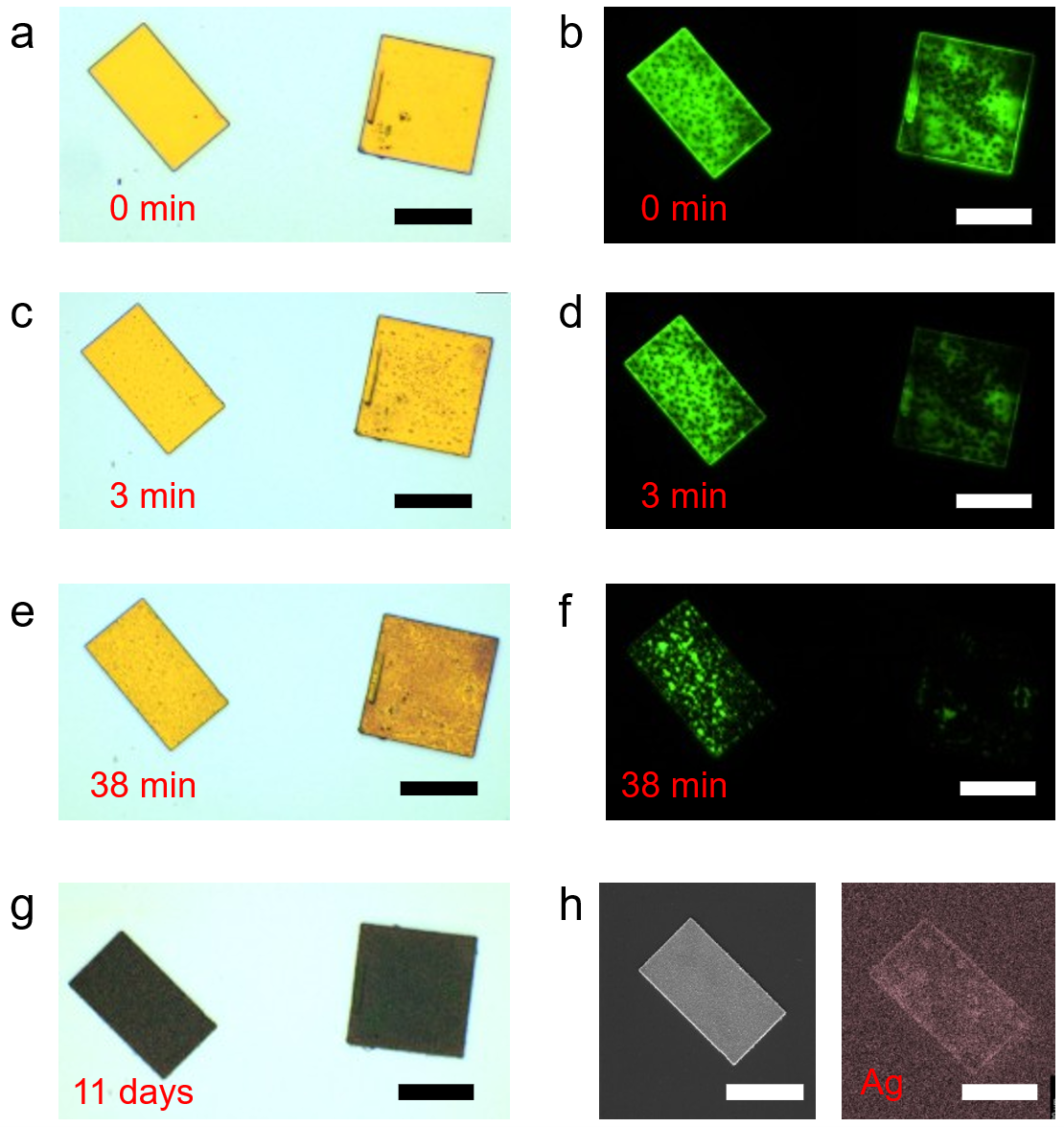


**Figure S10.** Optical microscope images and corresponding micro-PL images of the CsPbBr_3_ NPs on a silver thin film stored for **(a-b)** 0 min, **(c-d)** 3 min, **(e-f)** 38 min, **(g)** 11 days in nitrogen atmosphere. **(h)** SEM image of CsPbBr_3_/Ag stored for 11 days in a nitrogen atmosphere and its EDS mapping of Ag element. Scale bars: 5 μm.


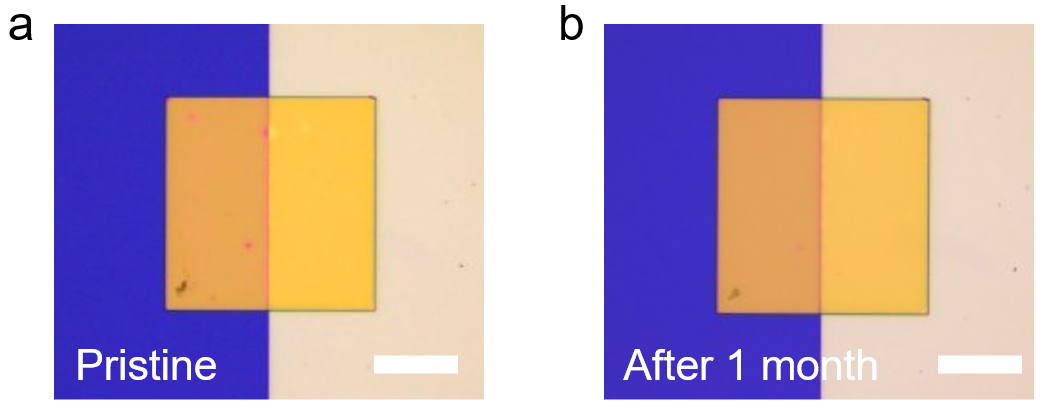


**Figure S11.** Optical microscope images of a CsPbBr_3_ NP partially in contact with a gold thin film in pristine state **(a)** and after stored in nitrogen environment for one month **(b)**. Scale bars: 5 μm.


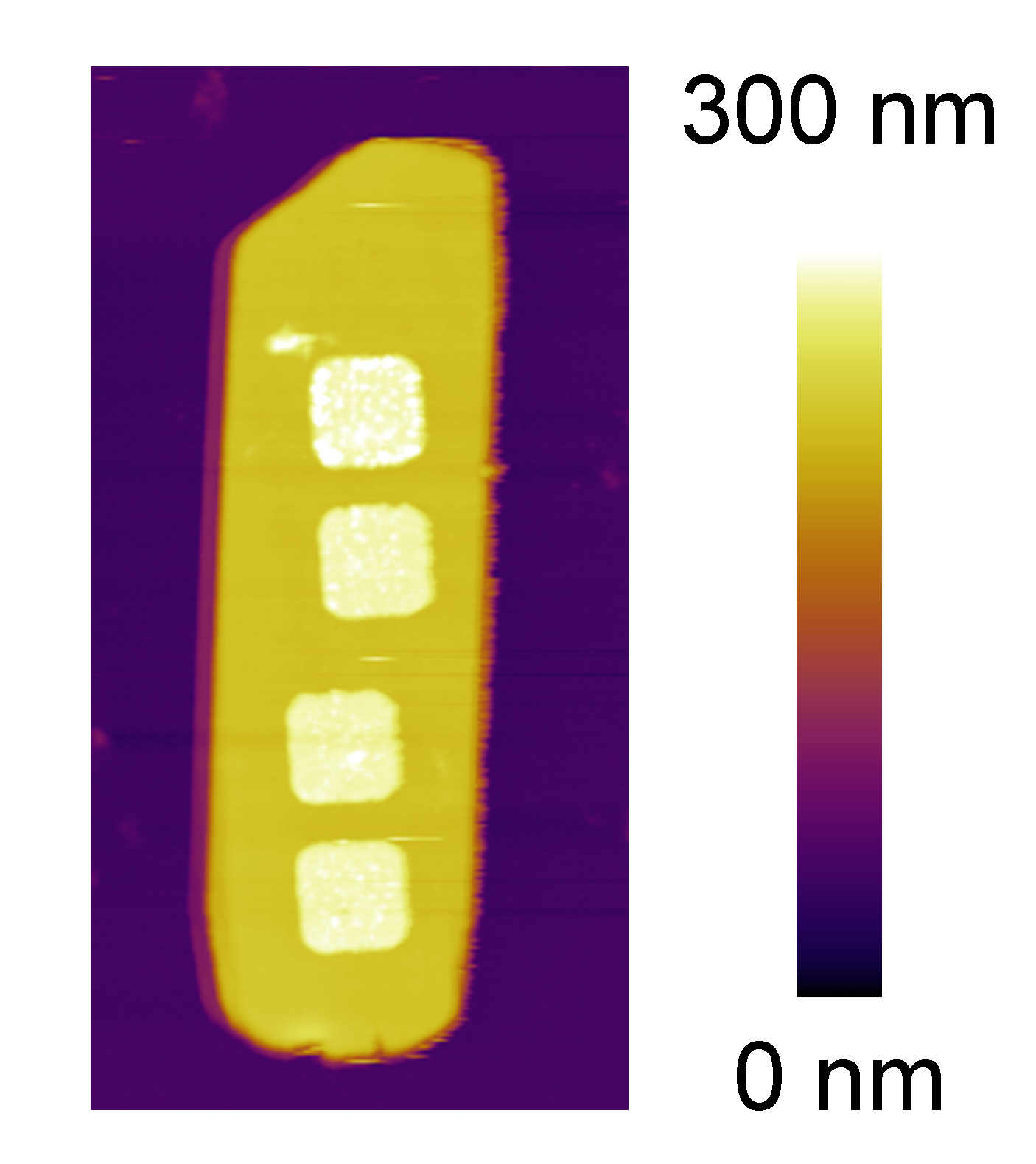


**Figure S12.** AFM image of four Au/CsPbBr_3_/Au devices with 1.5 × 1.5 μm TE. The top device was subjected to multiple voltage sweeps. Scale bar: 2 μm.


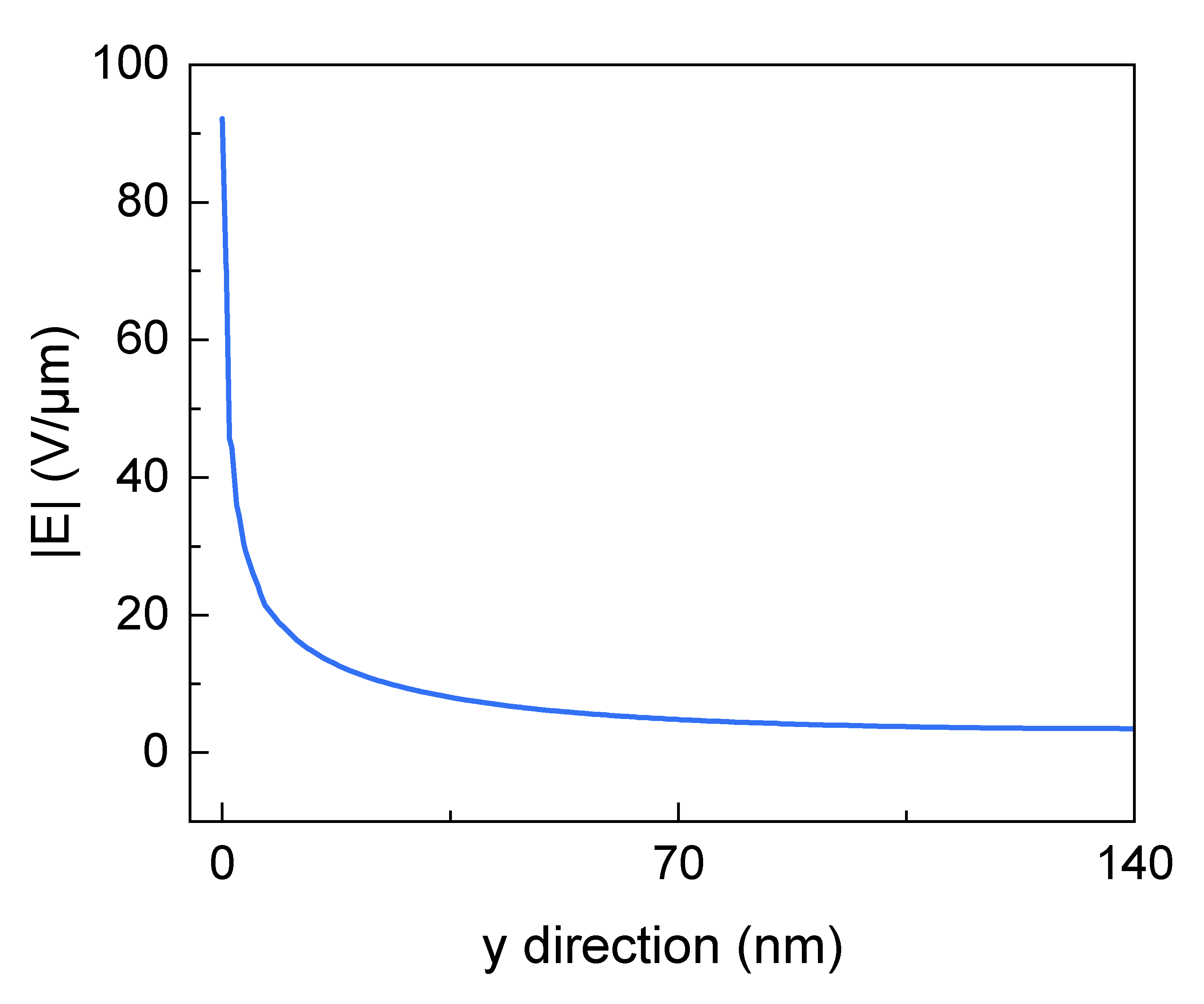


**Figure S13.** |E| profile along the vertical direction (y-axis) at the edge of TE.


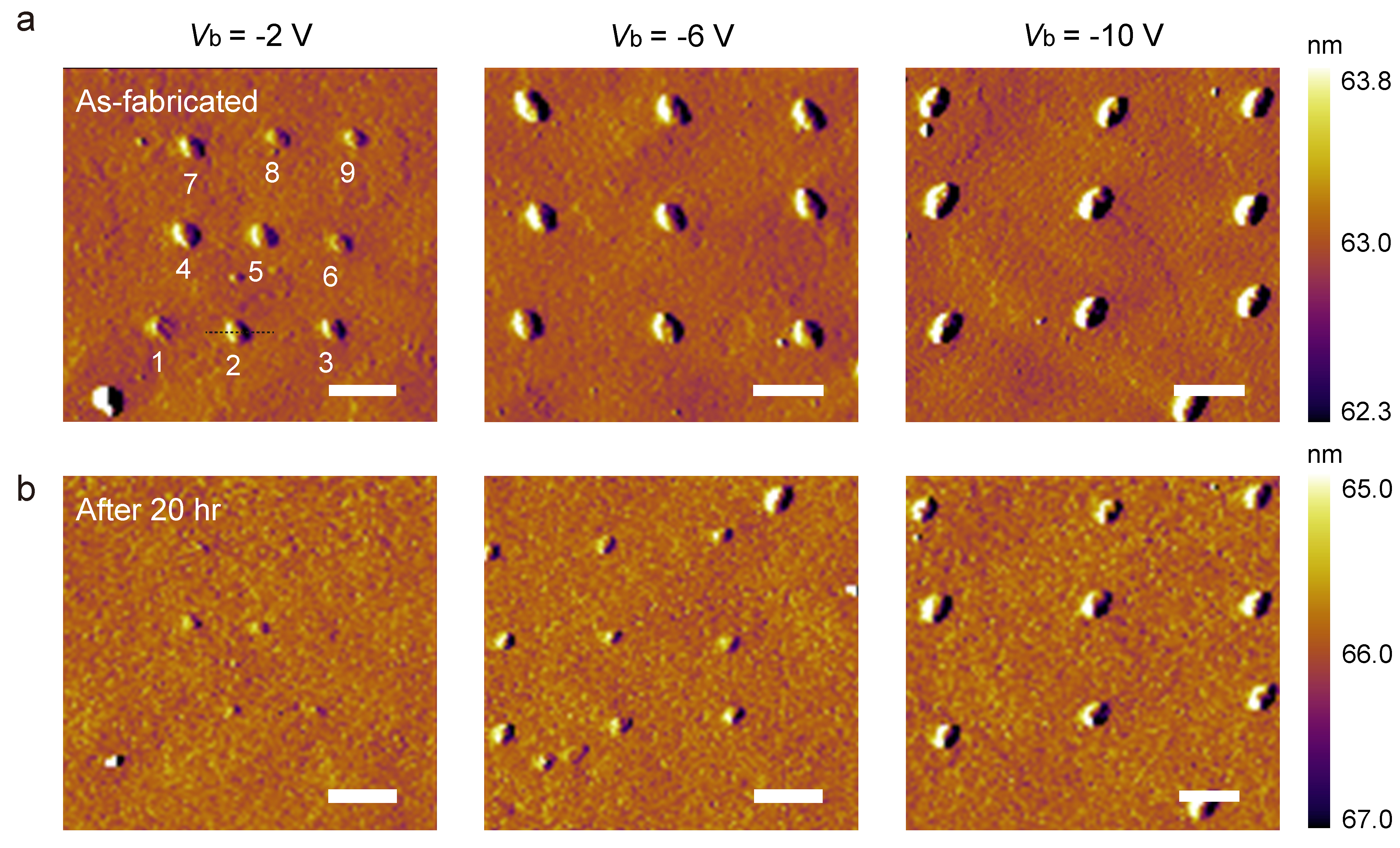


**Figure S14.** Amplitude images corresponding to the height images in Figure 5. **(a)** Amplitude images of 3 × 3 protrusion arrays fabricated by voltage sweeps to -2 (left), -6 (middle) and -10 V (right). **(b)** Amplitude images of (a) after storage for 20 hours in a N_2_-filled glove box. All the scale bars are 500 nm.


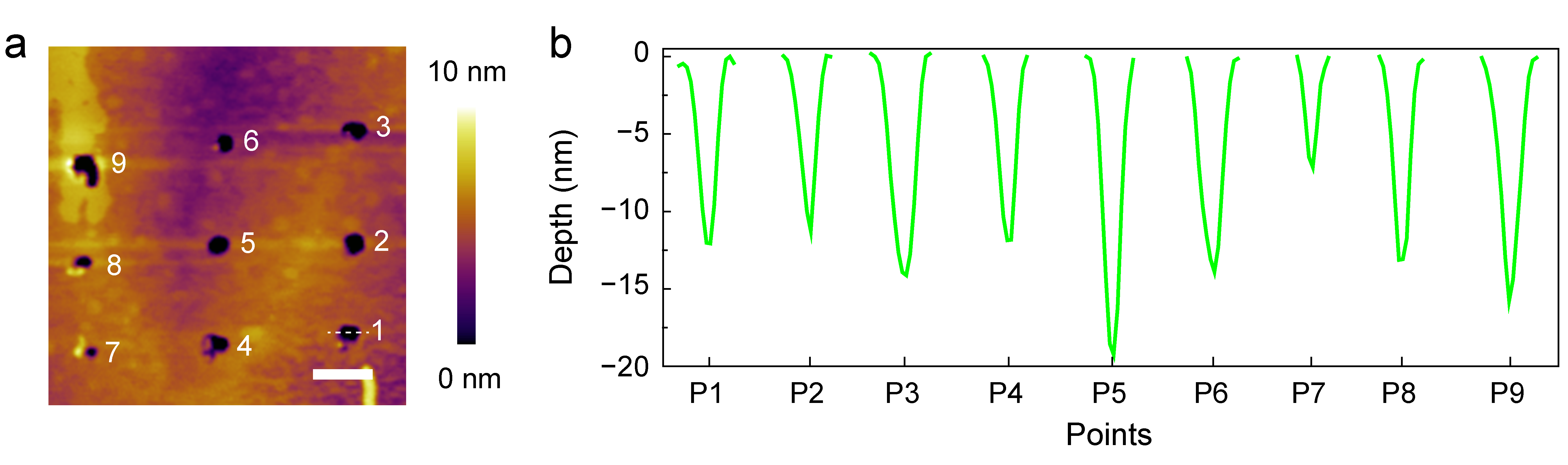


**Figure S15.** AFM image of the dents after positive voltage sweeps. **(a)** The 3 × 3 arrays of dents after positive voltage sweeps of +3 V. Scale bar: 200 nm. **(b)** Local height profiles at each location of the dent arrays.


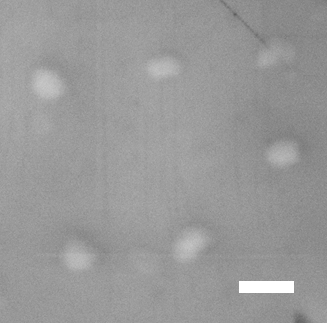


**Figure S16.** SEM image of the surface bumps stimulated under -10 V voltage sweep and after storage for 10 days in a N_2_-filled glove box. Scale bar: 500 nm.


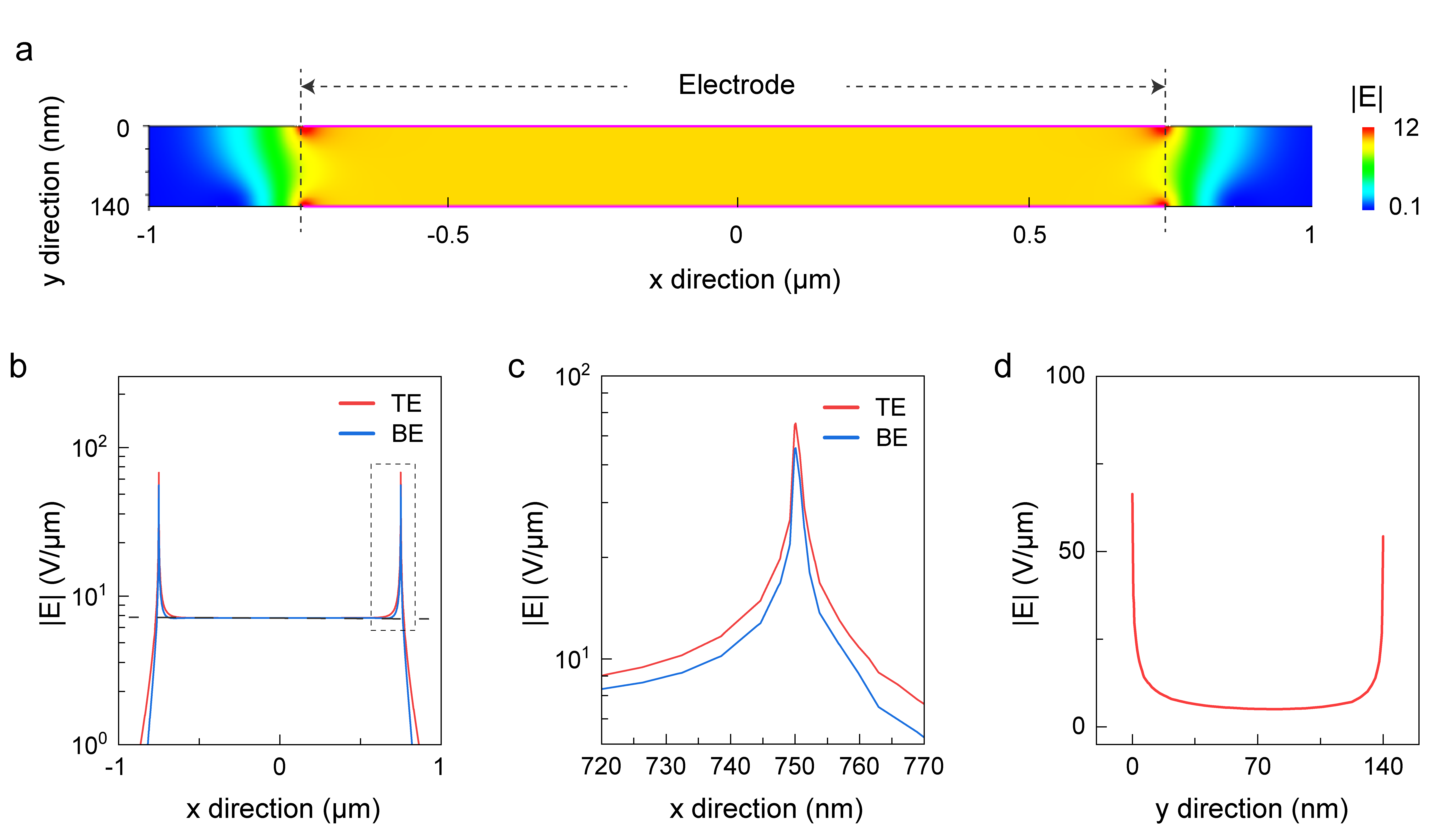


**Figure S17.** Simulated electric field distribution in a crossbar structure with microscale electrodes configuration. **(a)** Simulated distribution of the electric field strength. The width of both TE and BE is 1.5 μm, and the semiconductor layer is 50 μm. The unit for |E| is V/μm. **(b)** |E| profiles at the top and bottom interface at bias of 1 V. **(c)** Magnified view of |E| from the black dashed box in (b). **(d)** |E| profile along the vertical direction (y-axis) at the edge of TE.


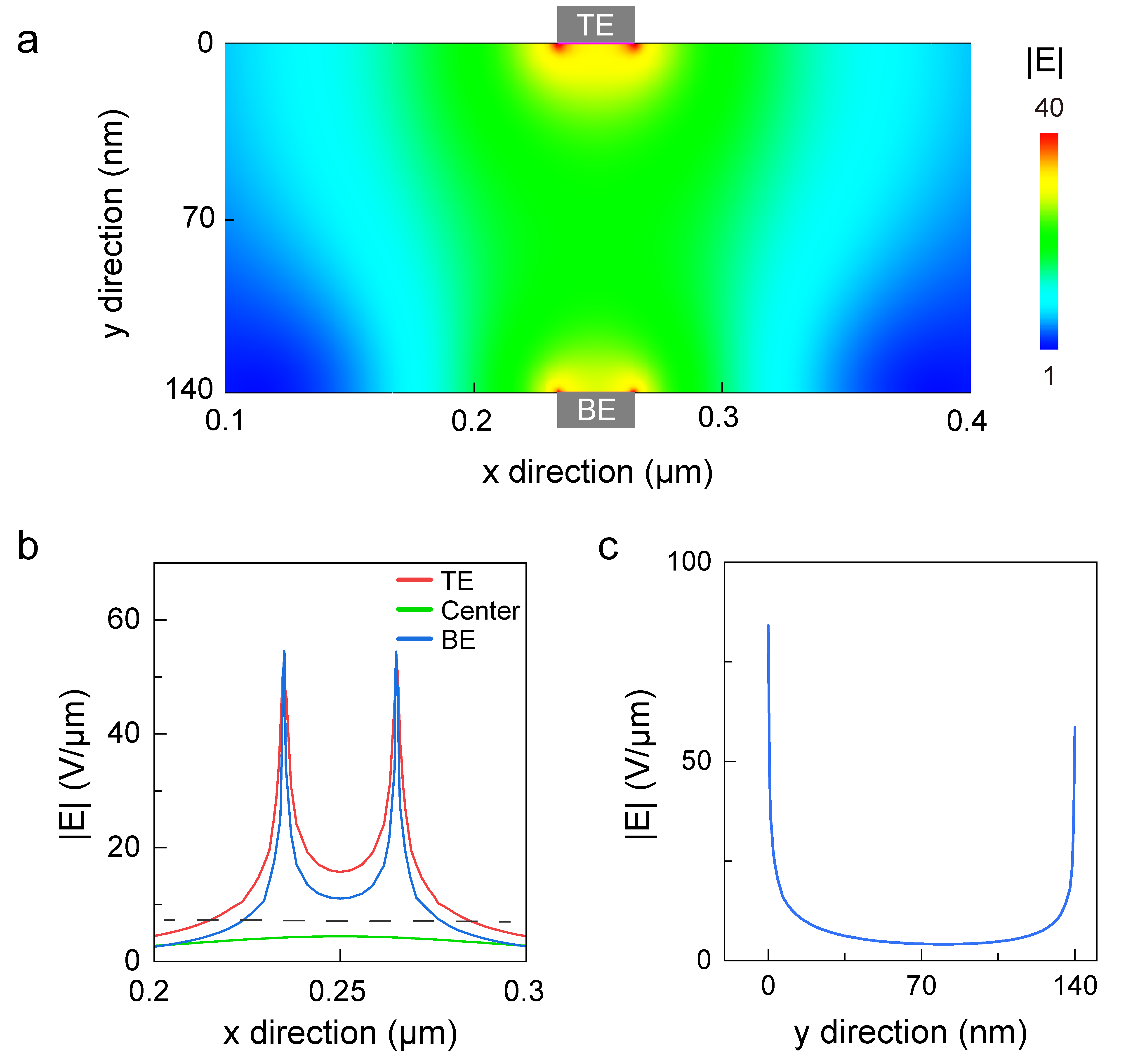


**Figure S18.** Simulated electric field distribution in a crossbar structure with nanoscale electrodes configuration. **(a)** Simulated distribution of electric field strength for TEs and BEs with 30 nm width. The semiconductor layer is 3 μm. The unit for |E| is V/μm. **(b)** |E| profiles across the top and bottom interface at bias of 1 V. **(c)** |E| profile in the vertical direction at the edge of TE.

**References**

[1] L. T. Li, Y. Chen, C. M. Cai, P. P. Ma, H. Y. Ji, G. F. Zou, *Small* **2022**, 18, 2103881.

[2] K. Kang, H. Ahn, Y. Song, W. Lee, J. Kim, Y. Kim, D. Yoo, T. Lee, *Adv. Mater.* **2019**, 31, 1804841.

[3] J. S. Han, Q. V. Le, J. Choi, K. Hong, C. W. Moon, T. L. Kim, H. Kim, S. Y. Kim, H. W. Jang, *Adv. Funct. Mater.* **2018**, 28, 1705783.

[4] J. Y. Seo, J. Choi, H. S. Kim, J. Kim, J. M. Yang, C. Cuhadar, J. S. Han, S. J. Kim, D. Lee, H. W. Jang, N. G. Park, *Nanoscale* **2017**, 9, 15278.

[5] S. J. Kim, I. H. Im, J. H. Baek, S. H. Park, J. Y. Kim, J. J. Yang, H. W. Jang, *ACS Nano* **2024**, 18, 28131.

[6] Z. H. Liu, P. P. Cheng, R. Y. Kang, J. Zhou, X. Zhao, J. Zhao, Z. Y. Zuo, *Adv. Mater. Interfaces* **2023**, 10, 2201513.

[7] F. Cao, Z. J. Hu, T. T. Yan, E. Hong, X. L. Deng, L. M. Wu, X. S. Fang, *Adv. Mater.* **2023**, 35, 2304550.

[8] S. Y. Kim, D. A. Park, N. G. Park, *ACS Appl. Electron. Mater.* **2022**, 4, 2388.

[9] F. F. Luo, Y. Z. Wu, J. W. Tong, F. B. Tian, X. M. Zhang, *Nano Res.* **2023**, 16, 10108.

[10] Y. C. Zheng, D. F. Yu, H. J. Lian, H. Y. Yuan, Y. Zhou, S. Yang, *Sci. China Mater.* **2023**, 66, 2383.

[11] X. F. Cao, Z. Z. Ma, T. Cheng, Y. D. Wang, Z. F. Shi, J. Z. Wang, L. Zhang, *Energy & Environ. Mater.* **2023**, 6, e12419.

[12] F. F. Luo, Y. Z. Wu, J. W. Tong, G. W. Qin, X. M. Zhang, *J. Alloys Compd.* **2023**, 938, 168498.

[13] X. Q. Ma, J. J. Zhou, Y. L. Liu, S. A. Xu, S. K. Cao, *Small* **2023**, 19, 2206852.

[14] I. H. Im, S. J. Kim, J. H. Baek, K. J. Kwak, T. H. Lee, J. W. Yang, D. E. Lee, J. Y. Kim, H. R. Kwon, D. Heo, S. Y. Kim, H. W. Jang, *Adv. Funct. Mater.* **2023**, 33, 2211358.

[15] J. Chen, D. J. Morrow, Y. Fu, W. Zheng, Y. Zhao, L. Dang, M. J. Stolt, D. D. Kohler, X. Wang, K. J. Czech, M. P. Hautzinger, S. Shen, L. Guo, A. Pan, J. C. Wright, S. Jin, *J. Am. Chem. Soc* **2017**, 139, 13525.

[16] J. Liu, F. J. Liu, H. N. Liu, R. Hou, J. Y. Yue, J. Z. Cai, Z. S. Peng, J. Impundu, L. M. Xie, Y. J. Li, L. F. Sun, *Small* **2020**, 16, 1906185.

[17] Y. Bao, H. Wang, M. An, H. Tang, J. Li, J. Li, C. Tan, Y. Luo, J. Xu, Y. Yang, *Nano Res.* **2024**, 17, 4610.

[18] H. Wang, Y. Bao, J. Li, D. Li, M. An, L. Tang, J. Li, H. Tang, Y. Chi, J. Xu, Y. Yang, *J. Phys. Chem. Lett.* **2023**, 14, 9943.
